# Transcriptomic analysis reveals novel downstream regulatory motifs and highly transcribed virulence factor genes of *Entamoeba histolytica*

**DOI:** 10.1186/s12864-019-5570-z

**Published:** 2019-03-12

**Authors:** Sarah Naiyer, Devinder Kaur, Jamaluddin Ahamad, Shashi Shekhar Singh, Yatendra Pratap Singh, Vivek Thakur, Alok Bhattacharya, Sudha Bhattacharya

**Affiliations:** 1 School of Environmental Sciences, Jawaharlal Nehru University, New Delhi, India; 2 Centre for Systems Biology, School of Life Sciences, University of Hyderabad, Hyderabad, India; 3 School of Life Sciences, Jawaharlal Nehru University, New Delhi, India

**Keywords:** *Entamoeba histolytica*, Transcriptome, Downstream promoter motif, Virulence genes, Serum starvation, Differential expression, Motif enrichment, Highly transcribed genes

## Abstract

**Background:**

Promoter motifs in *Entamoeba histolytica* were earlier analysed using microarray data with lower dynamic range of gene expression. Additionally, previous transcriptomic studies did not provide information on the nature of highly transcribed genes, and downstream promoter motifs important for gene expression. To address these issues we generated RNA-Seq data and identified the high and low expressing genes, especially with respect to virulence potential. We analysed sequences both upstream and downstream of start site for important motifs.

**Results:**

We used RNA-Seq data to classify genes according to expression levels, which ranged six orders of magnitude. Data were validated by reporter gene expression. Virulence-related genes (except AIG1) were amongst the highly expressed, while some kinases and BspA family genes were poorly expressed. We looked for conserved motifs in sequences upstream and downstream of the initiation codon. Following enrichment by AME we found seven motifs significantly enriched in high expression- and three in low expression-classes. Two of these motifs (M4 and M6) were located downstream of AUG, were exclusively enriched in high expression class, and were mostly found in ribosomal protein, and translation-related genes. Motif deletion resulted in drastic down regulation of reporter gene expression, showing functional relevance. Distribution of core promoter motifs (TATA, GAAC, and Inr) in all genes revealed that genes with downstream motifs were not preferentially associated with TATA-less promoters. We looked at gene expression changes in cells subjected to growth stress by serum starvation, and experimentally validated the data. Genes showing maximum up regulation belonged to the low or medium expression class, and included genes in signalling pathways, lipid metabolism, DNA repair, Myb transcription factors, BspA, and heat shock. Genes showing maximum down regulation belonged to the high or medium expression class. They included genes for signalling factors, actin, Ariel family, and ribosome biogenesis factors.

**Conclusion:**

Our analysis has added important new information about the *E. histolytica* transcriptome. We report for the first time two downstream motifs required for gene expression, which could be used for over expression of *E. histolytica* genes. Most of the virulence-related genes in this parasite are highly expressed in culture.

**Electronic supplementary material:**

The online version of this article (10.1186/s12864-019-5570-z) contains supplementary material, which is available to authorized users.

## Background

Advances in transcriptomics have provided the means to obtain detailed information about the entire gene expression program of a cell, under different growth conditions, environmental stresses, and differentiation pathways. For non-model organisms not amenable to genetic manipulation this is a particularly valuable tool in gene expression studies. *Entamoeba histolytica*, a protist, is the causative agent of amoebiasis that continues to be a major public health problem in the developing world [1]. The dormant, non-motile cyst is the infective form, which upon ingestion gets converted to the actively dividing trophozoite in the colon. In the majority of infections, the trophozoites get reconverted to cysts which are excreted in the feces, and the host remains asymptomatic. However, in some instances the trophozoites invade the intestinal mucosa, resulting in rapid tissue damage. The infection can also spread to other organs, notably the liver, causing liver abscesses.

Transcriptomics has been used in a number of studies to understand the gene expression changes in trophozoite to cyst conversion, and between *E. histolytica* strains differing in virulence potential, or grown under various environmental stresses [2–9]. The genes significantly modulated during human colon invasion included those in metabolic processes, glycosylases, and those related to cytoskeleton and DNA repair activities [10]. During encystation, many genes were up regulated relative to trophozoites at early stages of encystation while at later stages more genes were down regulated. There was general down-regulation of genes involved in metabolic processes as cysts matured; and transcription of these genes resumed during excystation [11]. The genes up-regulated at early and late stages of encystation included those encoding transporters, cytoskeletal proteins, vesicular trafficking, Myb transcription factors, cysteine proteases, components of the proteasome, and chitin biosynthesis [8].

While these transcriptome-level studies provide a glimpse of important changes in gene expression programs, transcriptome data have also been used to study the nature of *E. histolytica* gene promoters [12], alternative splicing and polyadenylation sites, and identification of novel coding transcripts [13]. In a study on genome-wide transcriptional regulatory patterns, Hackney et al. [12] identified one promoter motif associated with high gene expression, and three motifs associated with low expression. Presence of ≥2 of the latter motifs was predictive of low expression. Hon et al. [13] found pervasive alternative polyadenylation and splicing in *E. histolytica* and quantified the extent of stochastic noise in these alternative events. They found the functional impact of these processes to be limited to a small proportion of genes, with most of the microheterogeneity likely to arise from stochastic events.

Some of the important issues not covered in the previous studies include information on the nature of highly transcribed genes of *E. histolytica,* and role of any downstream promoter motifs in gene expression. In the study to identify promoter motifs associated with gene expression levels [12] only upstream sequences were analysed, and microarray expression data were used, which typically have a much lower dynamic range of gene expression levels than RNA-Seq data generated by high throughput sequencing [14]. There is also paucity of information regarding the normal expression status of *E. histolytica* genes coding for known virulence factors. We have generated RNA-Seq data from *E. histolytica* trophozoites grown under normal lab conditions and those subjected to growth-stress by serum starvation and recovery after serum replenishment. We have analysed sequences flanking the putative start codons of genes belonging to high and low expression classes and report novel downstream promoter motifs associated with high expression. The functional relevance of some of these motifs was experimentally validated by reporter gene expression. Interestingly, the highly transcribed genes included virulence factors like the amoebapores, Gal/GalNAc lectin (light subunit), and some calcium-binding proteins and cysteine proteases.

## Results

### RNA-Seq analysis

Poly (A)-enriched RNA was obtained from three sets of *E. histolytica* trophozoites- 1) grown under normal culture conditions; 2) subjected to serum starvation for 24 h; 3) replenished with serum for 2 h, following starvation. cDNA libraries were prepared from two biological replicates of each condition, and analysed using paired-end, high-throughput Illumina sequencing. A total of 426,458,460 RNA reads of average size 100 bp were generated from the 6 samples, and > 90% reads in all samples were uniquely mapped to the genome of *E. histolytica* (see Additional file 1)*.* The quality of RNA-Seq data was evaluated by read distribution, 5′/3′ bias and sequencing depth. Of total 8333 annotated transcripts in *E. histolytica*, we could detect expression of 7962 (96%) transcripts in our RNA-Seq data, while 371 showed zero transcripts per million (TPM). About 89% reads mapped to exonic regions, with only 0.06% reads from introns owing to their low abundance in *E. histolytica* [15]. The remaining 11% reads aligned to intergenic regions, which would also include transcripts from repeat elements (see Additional file 2a). Reads were uniformly distributed throughout the gene body and there was no obvious 5′/3′ bias (see Additional file 2b).

The sequencing depth of RNA-Seq data can be checked by looking at saturation plot for annotated features like splice junctions. These junctions can be predetermined from reference gene model. The sequencing depth of our RNA seq data was almost saturated for “known junction” and rapidly reached a plateau (see Additional file 2c). We further looked for novel splice junctions. Of the total splice junctions detected by RNA-Seq data, 73% were found to be novel, which included 60% complete novel, and 13% partial novel junctions (see Additional file 2d). However, when we checked for the occurrence of actual splicing events (not predicted splicing junctions), just 10% appeared to be novel, indicating that most of the apparently novel splice junctions were not used (see Additional file 2e). The existence of a large number of un-annotated junctions, and presence of inactive novel splice junctions from RNA-Seq data has already been described [13], and our results were in line with this data. Comparison of log_2_ TPM values showed that our two biological replicates for each growth condition were in close agreement with one another having a Pearson’s correlation coefficient of 0.97 (for Normal), 0.96 (for Serum starved) and 0.98 (for Serum replenished) (see Additional file 3). These observations confirm the overall high quality of our sequencing data. Quantification of gene expression using TPM values showed that under normal growth conditions the range of expression varied over 5 × 10^6^ fold from the highest log_2_ TPM of 14.6 to the lowest log_2_ TPM of − 7.6. Such a range of gene expression was also reported in transcriptome data of *Saccharomyces cerevisiae* [16]. With less sensitive SAGE data the range of expression covered three orders of magnitude due to poorer detection of low-abundance transcripts [17]. We classified gene sets based on average log_2_ TPM, which is described later.

### Experimental validation of transcriptome data

**Fig. 1:**
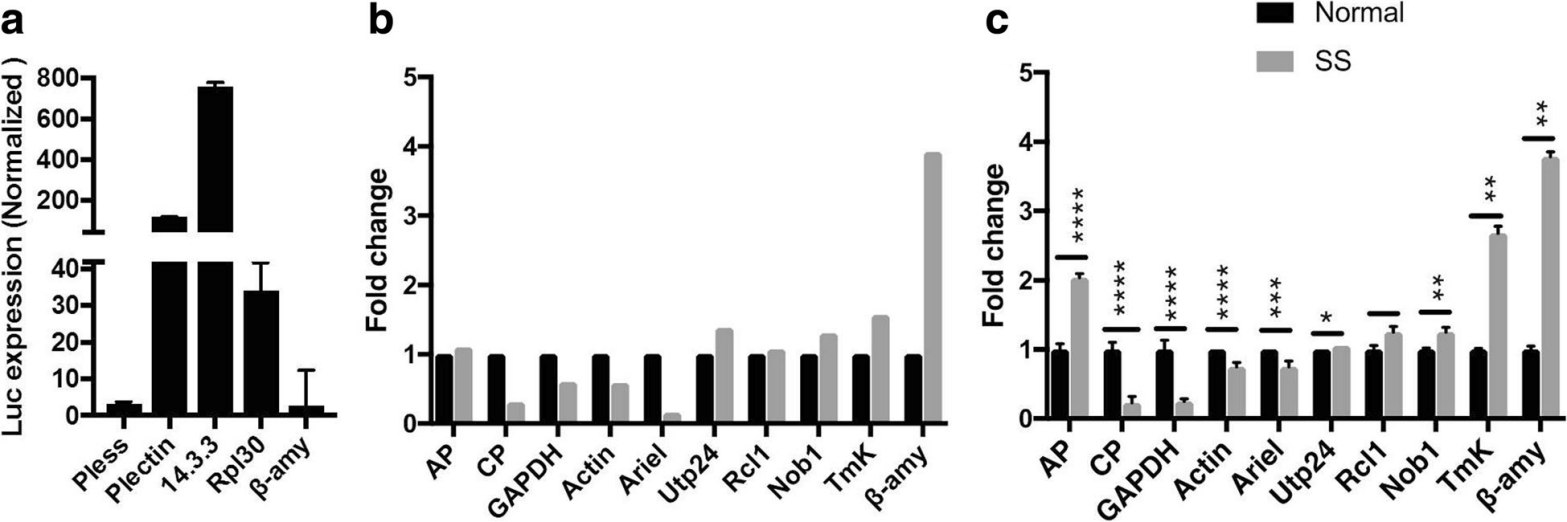
**a**) Luciferase reporter assay in transfectants containing the regulatory sequence from a very high expressed gene (14–3-3), medium expressed gene (Rpl30) and low expressed gene (ß-amylase). **b**) Fold change of expression values of selected genes in normal and serum starved cells from the transcriptome data from RNA Seq analyses; **c**) Real time PCR data of the same genes, showing similar trend as RNA seq. Data. AP- amoebapore, CP- cysteine protease, TmK- TMKB1–18, β-amy- β-amylase, SS- serum starvation

We selected three genes with high, medium or low average TPM values as obtained from RNA-Seq data, and measured luciferase reporter expression driven by each of these gene promoters. The selected genes were 14–3-3 (EHI_006810) (high expression, TPM 720), Rpl30 (EHI_192800) (medium expression, TPM 296), and ß-amylase (EHI_148800) (low expression, TPM 1.58). DNA sequences flanking the AUG start codon (400 bp upstream and 100 bp downstream) of each selected gene were cloned in pEhNeoluc vector of *E. histolytica* (see Additional file 4) upstream of luciferase reporter gene. Stably expressing *E. histolytica* cell lines were obtained by transfection, and the expression of luc (luciferase) was measured. The 14–3-3 gene promoter showed the highest expression of luc, followed by promoters of Rpl30 and ß-amylase (Fig. 1a). This corroborated very well with the RNA-Seq data for these genes. Further, to validate the differential expression during serum starvation we selected ten genes spanning the range of expression from high to low (Fig. 1b, c). Their expression under normal and serum starved conditions was determined by qRT- PCR taking 18S rRNA as an endogenous control (dilution 1:100). TMKB1–18 was also used as a reference since this gene is known to be up regulated 1.8-fold during serum starvation in *E. histolytica* [18]. The RNA-Seq data (Fig. 1b) matched well with the real time expression data (Fig. 1c), and the fold change showed similar trend in both cases, although the absolute fold-change values were different for some genes.

### Classification of gene sets on the basis of expression

**Fig. 2:**
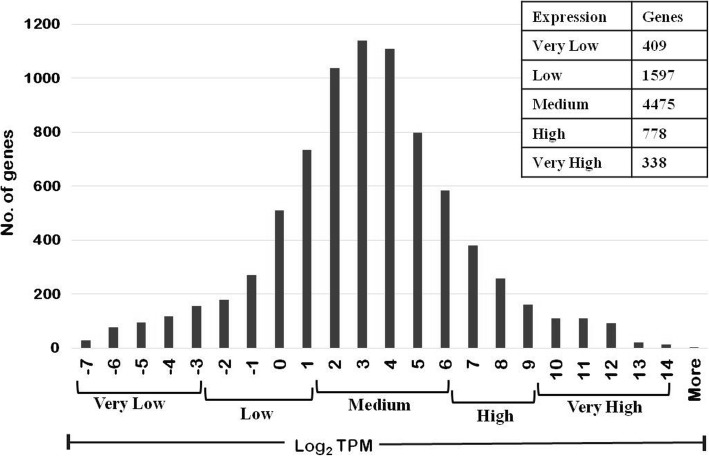
Classification of genes based on log_2_ normalized TPM: The log_2_ TPM values ranged from −7 to + 14. These were arbitrarily divided into expression classes as shown

We used RNA-Seq data to classify the genes according to expression levels in normal growth conditions. Of the 7962 transcripts with non-zero TPM we removed those where a minimum genomic stretch of 400 bp was not available upstream of the AUG start codon, or which contained stretches of N. 365 such sequences were removed. The remaining 7597 transcripts were used for further analysis. A plot of the Log_2_ TPM values showed a normal distribution (Fig. 2). The distribution was split into 5 groups with increasing expression levels: Very High (VH) (≥9), High (H) (6 to < 9), Medium (M) (1 to < 6), Low (L) (− 3 to < 1) and Very Low (VL) (<− 3 to − 7.6). The number of genes in each class were- 338 (VH), 778 (H), 4475 (M), 1597 (L), and 409 (VL).

### Functional categories of high and low expressing genes

**Fig. 3:**
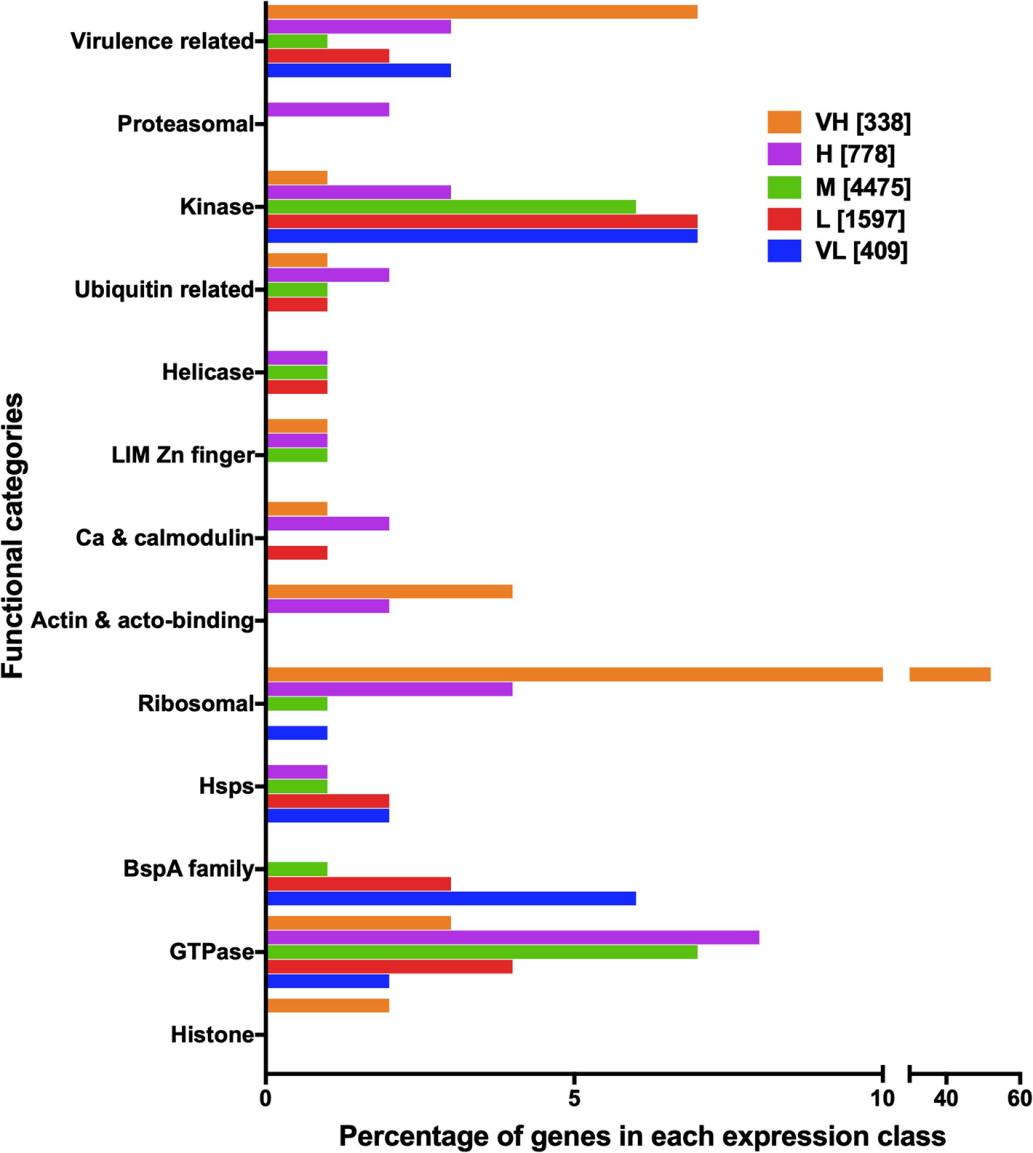
Distribution of genes of different functional categories in each expression class

We checked whether the high or low expressing genes were associated with particular functional categories. Based on Gene Ontology (GO) analysis (see Additional file 5) and annotation in the database the major functional categories associated with each expression class was determined (see Additional files 6 and 7]. The data are summarized in Table 1 and shown graphically in Fig. 3.

**Table 1:** Genes of major functional categories in each expression class

Functional category	No. of members	Expression (%)				
		VH [338]	H [778]	M [4475]	L [1597]	VL [409]
Virulence related	138	25 (7.40)	22 (2.82)	52 (1.16)	28 (1.75)	11 (2.69)
GTPase	444	10 (2.96)	61 (7.83)	296 (6.63)	68 (4.26)	9 (2.2)
BspA family	124	–	1 (0.13)	50 (1.12)	49 (3.07)	24 (5.87)
Hsps	94	–	4 (0.51)	58 (1.3)	24 (1.5)	8 (1.96)
Ribosomal	276	175 (51.78)	35 (4.49)	58 (1.3)	4 (0.25)	4 (0.98)
Actin & acto-binding	52	13 (3.85)	17 (2.18)	19 (0.43)	2 (0.13)	1 (0.24)
Histone	17	6 (1.78)	3 (0.39)	7 (0.16)	1 (0.06)	–
Ca & calmodulin	52	3(0.89)	15 (1.93)	21 (0.47)	12 (0.75)	1 (0.24)
LIM Zn finger	18	4 (1.18)	7 (0.9)	6 (0.13)	–	1 (0.24)
Helicase	63	–	4 (0.51)	38 (0.85)	19 (1.19)	2 (0.49)
Proteasomal	35	–	16 (2.05)	18 (0.4)	–	1 (0.24)
Ubiquitin related	64	3 (0.89)	14 (1.8)	32 (0.72)	14 (0.88)	1 (0.24)
Kinase	432	4 (1.18)	21 (2.7)	273 (6.11)	104 (6.51)	30 (7.33)

Legend: Total number of genes in each expression class is in bold square brackets

### Values in parenthesis are percent of genes in that expression class

The medium expression class included a very large number of genes; hence it had representatives from each functional category. Excluding this class, it was possible to derive information about association of expression levels with functional categories. Highly expressing gene categories (with relatively poor representation in L and VL classes, and good representation in H and VH classes) were ribosomal, actin, histone, proteasomal, and LIM domain-containing proteins. Conversely, the low expressing gene categories were BspA family, Hsps, helicases and kinases. Further analysis of the VH expression class showed that of the 338 genes in this class, 44 were hypothetical. 175 genes (51.8%) represented the ribosomal protein and translation related genes. The remaining 119 genes belonged to various other functional classes (highly expressing genes listed above), and oxidative stress response genes (see Additional file 6). Interestingly, the second most abundant functional category (7.4%) in VH class belonged to genes implicated in amoebic virulence (Table 1). These genes included amoebapore A, B and C precursors, Gal/GalNAc lectin light subunit and a few of the cysteine proteases (CPs) (isoform EhCP-A5, considered as a virulence determinant [5]) (see Additional file 6). Calcium binding proteins are also important for amoebic virulence, being required for efficient phagocytosis [19, 20]. Some of the calcium binding protein genes were also amongst the VH class. High expression of oxidative stress response genes is important for this organism, being a microaerophile; and high expression of actin and cytoskeleton-related genes may be required to maintain the cytoskeleton of this actively phagocytic cell.

Of the 409 genes in VL expression class, 278 were hypothetical (see Additional file 7). A number of the kinases and BspA family genes (containing leucine rich repeats) were grouped in this class. Around 29 kinases and 24 BspA family genes were found. Other genes included some of the cysteine proteases, surface antigen Ariel1, AIG family proteins, DNA polymerase, regulator of nonsense transcripts, Hsps and cell metabolism genes. The presence of Hsps in this category is expected from cells growing optimally without stress. Low expressing genes were amongst those most commonly up regulated upon growth stress due to serum starvation. Low expressing genes like BspA family have been shown to be up regulated during oxidative stress in *E. histolytica* [21].

### Motif search in genes of different expression classes

**Fig. 4:**
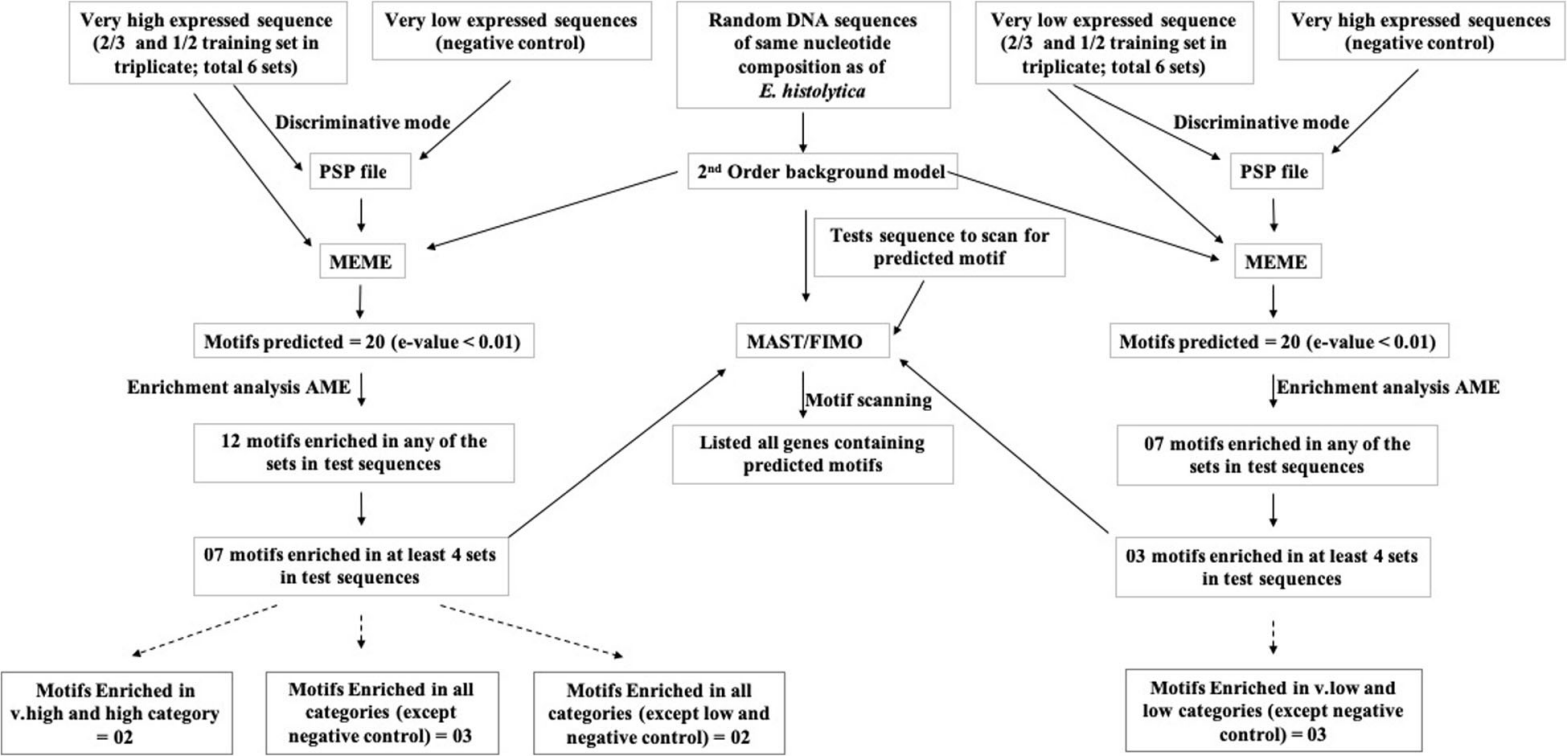
Flowchart to detect the motifs representative of high/low expression categories: To detect the motifs enriched in high expression class, the very low expression class was used as negative control. The reverse was done to search for motifs enriched in very low expression class. Of all the very highly expressed genes, two types of training sets were made, 2/3 and 1/2 of genes were used as training set and rest as test set. It was repeated in 3 rounds by randomly selecting genes for training and test set. The predicted motifs were checked for enrichment in test set and also in other expression classes (high, medium, and low)

Genes with similar steady state transcript levels could possess common regulatory elements, either in isolation or in a combinatorial fashion. An *in-silico* approach was used to identify such common sequence motifs in each expression class, as described in the flow chart (Fig. 4). We extracted 400 nt sequence upstream, and 100 nt downstream of putative AUG start codon of each gene for motif analysis in the 500 nt stretch. As described previously we could extract these sequences for 7597 of the 7962 expressed genes. We used discriminative MEME search version [22] as described in Methods. After enrichment analysis we obtained 12 motifs enriched in VH expression class. Five of these motifs (M7 and M12–15) were enriched with low probability. We did not consider them significant as they occurred in less than four of the six rounds of searching, and they were not further analysed. Seven motifs were significantly enriched based on *p*-values, and studied further (see Additional file 8) (Table 2).

**Table 2:** Predicted motifs enriched in genes of different expression classes

Motif	Sequence	E-value	Width	Expression class enriched	Position of motif	Ref [12]	
						Motif Number	Motif similarity (significance)
M1	AAAARRARARAA	2.2e-112	12	All	−50 to −25	M29	*p*-value 9.06e-02 E-value 5.44e-01
M2	AAAAAACCCTAD	1.1e-050	12	All	−50 to −25	M37	*p*-value 3.87e-05 E-value 2.32e-04
M5	GAACTAAAAAA	2.9e-028	11	All	−25 to −01	M40	*p*-value 2.63e-05 E-value 1.58e-04
M4	WGVWGBTGHTGV	2.9e-035	12	Very High	+ 25 to + 75	^a^M30	*p*-value 6.28e-04 E-value 3.77e-03
M6	TGVACCAAGAG	2.8e-014	11	Very High	+ 50 to + 100	–	–
M10	TYTTTTTCTTYT	5.1e-009	12	High, Medium	Dispersed	M35	*p*-value 8.96e-05 E-value 5.38e-04
M11	MATGKCWGVMR	2.4e-009	11	High, Medium	+ 1	–	–
L_M3	RAAWGAWGWWWA	4.8e-016	12	Low	Dispersed	M24	*p*-value 8.18e-04 E-value 4.91e-03
L_M4	TCATTCAWTS	1.3e-020	10	Low	+ 1	–	–
L_M9	WGATATTAATGA	3.0e-009	12	Low	+ 50	–	–

Legend: ^a^M30 was not considered to be significant in Hackney et al, [12]

These motifs were not significantly enriched in the VL expression class, which was used as negative control for the search. Most genes contained more than one motif, and many of the motifs occurred multiple times in the same gene. Promoter motifs predictive of gene expression levels in *E. histolytica* have earlier been reported by Hackney et al. [12]. Our study has yielded important new information as it was based on RNA-Seq analysis, using the latest annotated genome sequence of *E. histolytica* [15]. For enrichment analysis we used the AME tool which was added late in 2010 in MEME suite [23]. An important additional aspect of our work is that we looked for conserved motifs in sequences downstream of the initiation codon as well, rather than focusing only on the upstream sequences. Due to these factors our analyses showed some novel findings not reported earlier.

### Motifs associated with high gene expression

#### Motifs M1, M2 and M5

**Fig. 5:**
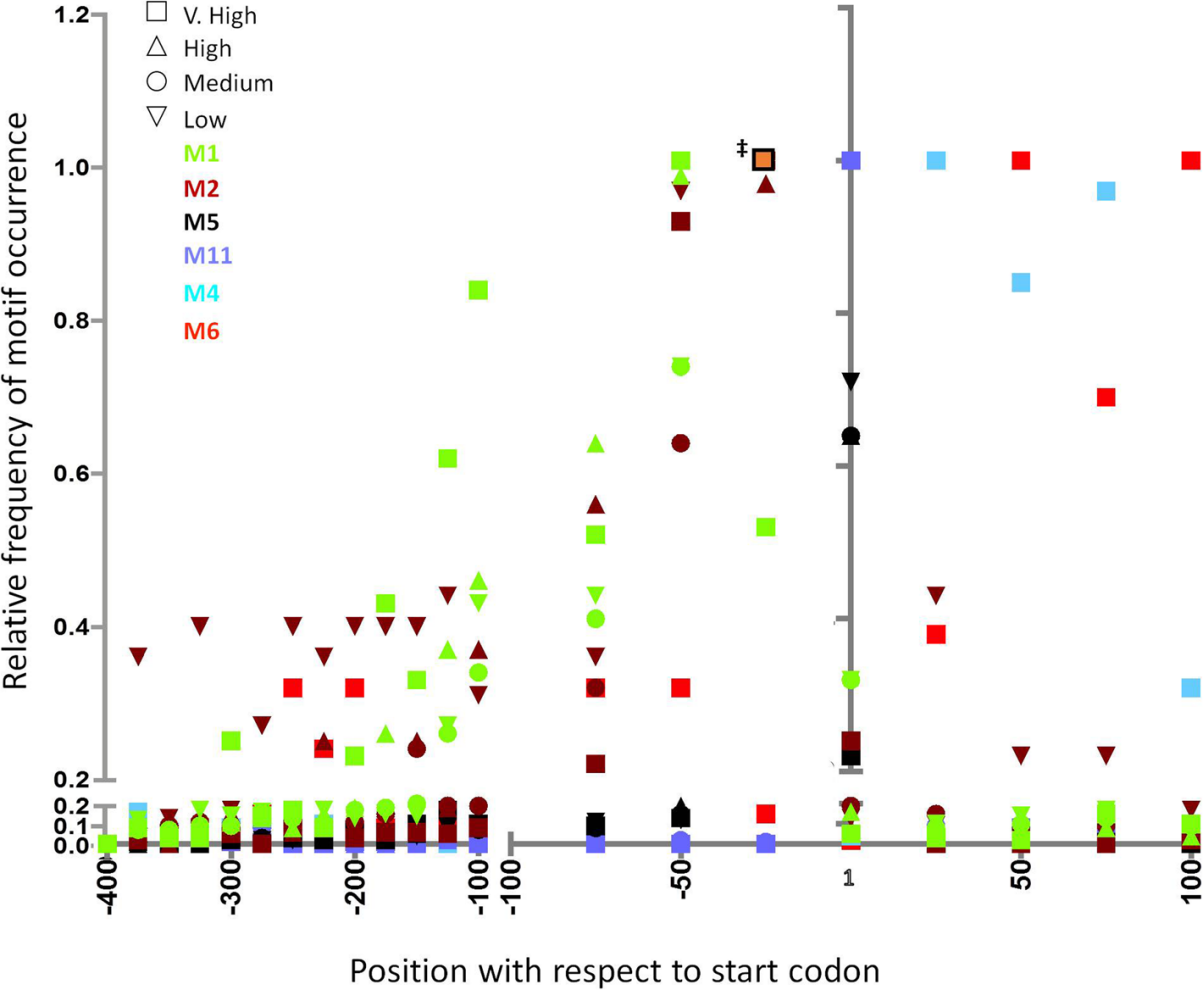
Relative enrichment of predicted motifs (M1, M2, M5, M11, M4, M6) in genes of various expression classes. Start codon is at position 1. ‡ Indicates most frequent occurrence of the following motifs: M1 High, M1 Medium, M1 Low, M2 V. high, M2 Medium, M2 Low, M5 V. high, M5 High, M5 Medium, M5 Low

These motifs were enriched in all the expression classes (except VL), and were highly A-rich. They were present upstream of AUG and their positions were conserved (Fig. 5) (see Additional file 9). These motifs have significant overlap with motifs reported by Hackney et al. [12] (Table 2). Motif M1 was found most commonly at − 50 to − 25 bp upstream of AUG start codon. However, it had a fairly high distribution further upstream as well. This motif was commonly found multiple times in the same gene. From the functional classification of genes according to GO terms this motif was most frequently seen in genes coding for small GTP-binding proteins, proteasome subunits, and translation-related proteins (see Additional file 5). Motif M2 was also most commonly located at positions − 50 to − 25, but compared with M1 it was less common further upstream, especially in the VH class. This motif was reported by Hackney et al. [12] as being predictive of high gene expression. In their analysis it was enriched in ribosomal protein genes and tRNA synthetase genes. We also found this motif in a large number of ribosomal protein and translation-related genes, in addition to RNA helicases and WD40 repeat-containing protein genes. Motif M5 was located at − 25 to − 1 with very few occurrences further upstream. It was commonly seen in ribosome and translation-related, GTP-binding and actin-related genes.

#### Motifs M4 and M6

These two motifs were exclusively enriched in VH expression class (*n* = 338). Motif M4 was predicted to be present in 132 genes whereas M6 was present in 63 genes. Interestingly, these motifs were mainly found downstream of AUG and were positionally conserved, with very few occurrences in the upstream regions. The motif M4 was present at + 25 to + 75 while M6 was present at + 50 to + 100 (Fig. 5) (Additional file 10). In the rare occurrence of these motifs in the negative control (VL expression class) their location was also different, as they were found dispersed upstream of AUG (Fig. 5) (Additional file 10). This is in accordance with the notion that both position and sequence of a motif determine its function. The sequence of M4 and M6 was not A-rich, although M6 was purine-rich, while M4 had greater sequence diversity. Since these motifs occurred in close proximity in the same gene, their physical overlap was checked by MAST, a tool from MEME suite [24]. In most genes we could not find a significant overlap indicating that the motifs were independent of each other. However, there were eight instances where these motifs overlapped, with significant *p*-value (≤0.01). Interestingly, all of these eight were actin gene sequences corresponding to transcriptionally active copies with TPM value > 1000 (see Additional file 11). A motif similar to M4 (M30) was also reported by Hackney et al. [12],. However, it was not considered significant in their study which included only upstream sequences. Analysis of the GO categories in which M4 and M6 motifs were found showed that they were highly enriched in ribosomal protein, and translation-related genes (see Additional file 5). In addition they were found in actin and actin-related genes. According to gene annotation they were also present in virulence-associated genes like amoebapore B, non-pathogenic pore forming peptide, and grainin genes (see Additional file 5). To our knowledge this is the first genome-wide report of downstream motifs in a protozoan parasite. Earlier studies have shown the presence of downstream promoter elements (DPEs) in the retrotransposon L1Tc of *Trypanosoma cruzi* [25], and in the pgs28 gene of *Plasmodium gallinaceum* [26]. DPEs were first discovered in the retrotransposon *jockey* of *Drosophila melanogaster* [27]. We checked for the presence of motifs M4 and M6 in the 5′-end (500 bp) of the most abundant *E. histolytica* retrotransposon EhLINE1 [28]. However, we could not find enrichment of these motifs in EhLINE1.

#### Motifs M10 and M11

These were enriched in high and medium expression classes. M10 was composed entirely of pyrimidines and was highly T-rich. This motif has also been reported earlier [12]. From GO analysis it was found in ribosomal protein genes, peroxiredoxin, histone fold, and nucleosome assembly genes. The position of M10 was not conserved; rather it was dispersed throughout the upstream region. The high T-richness of this motif could explain its wide distribution. Motif M11 showed greater sequence diversity than M10. It was positioned almost exclusively at the start codon, with only a few occurrences in downstream and upstream sequences (Fig. 5) (see Additional file 12). It was most commonly found in ribosomal protein genes, transmembrane proteins, thioredoxin, peroxiredoxin, and cell redox homeostasis genes.

### Motifs associated with low gene expression

The same procedure (Fig. 4) was used to look for motifs enriched in low expression class. We discovered 20 such motifs, using VH set as negative control. The motifs with e-value ≤0.01 were further analysed for motif enrichment using AME [23]. After motif enrichment we obtained 7 motifs specific for low expression. Of these, three motifs were enriched in VL and L classes in at least 4 rounds of training set. These motifs L_M3, L_M4 and L_M9 were further analysed (Table 2). All of these motifs were A + T-rich and showed poor positional conservation. Motif L_M3 was dispersed throughout the upstream region. Motif L_M4 was also seen throughout the upstream region but was enriched at the start codon. Motif L_M9 was dispersed throughout, but was comparatively enriched at + 50 position within the gene (see Additional file 13). Motif L_M3 was also reported by Hackney et al. (2007) as being predictive of low gene expression in combination with two other motifs. From GO analysis, motif L_M3 was found most frequently in protein kinase genes, dedicator of cytokinesis, and chaperonin Clp A/B genes (see Additional file 5).

### Frequency of motif co-occurrence

**Fig. 6:**
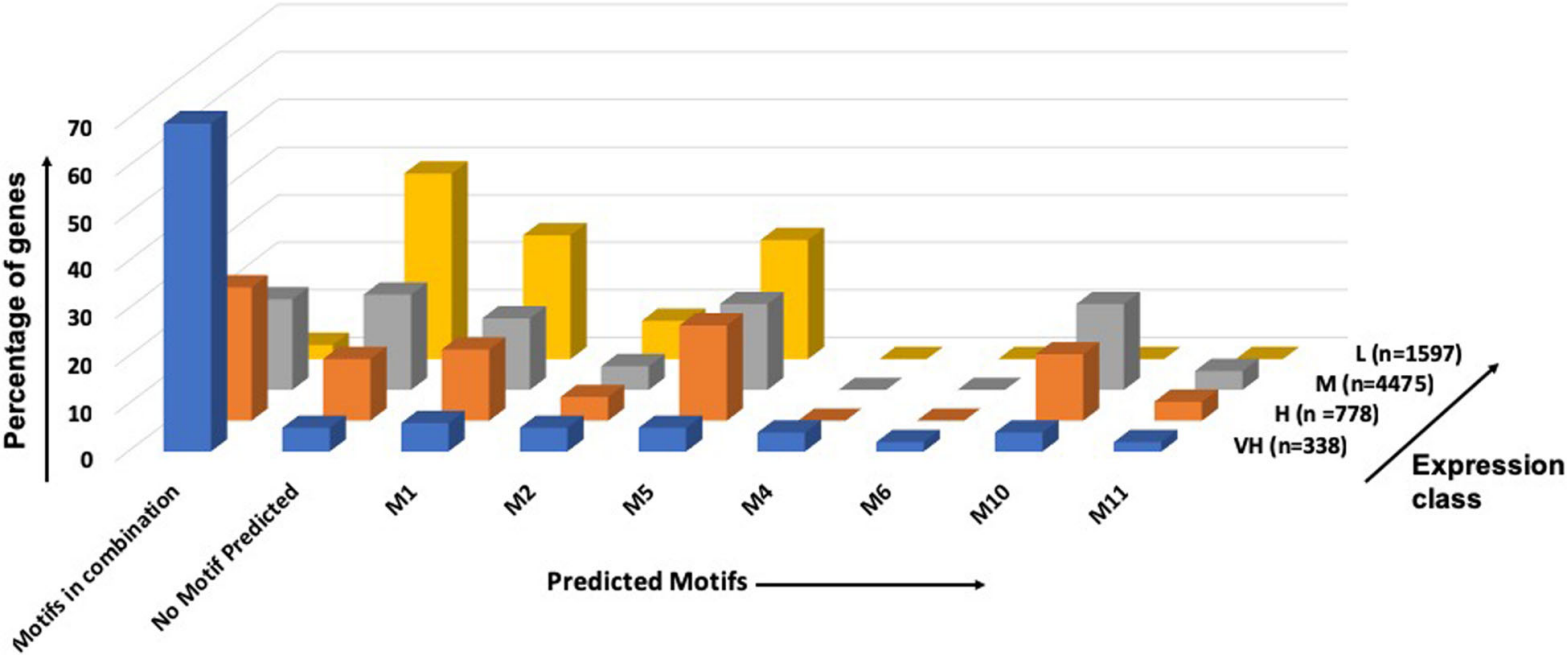
Percentage of genes in each expression class with the indicated motifs

The frequency of motif occurrence in each expression class and the number of genes in which each motif is found (alone or in combination) is shown in Table 3 and Fig. 6.

**Table 3:** Frequency of motifs in each expression class

Genes with Motifs	VH (n = 338)	H (*n* = 778)	M (*n* = 4475)	L (*n* = 1597)
M1 (Single occurrence)	19 (6%)	116 (15%)	684 (15%)	416 (26%)
M1 (All combinations)	188	406	1990	704
M1 (Average per gene)	1.58	1.48	1.23	1.15
M2 (Single occurrence)	16 (5%)	041 (05%)	234 (05%)	123 (08%)
M2 (All combinations)	154	133	558	164
M2 (Average per gene)	0.55	0.21	0.13	0.11
M5 (Single occurrence)	16 (5%)	154 (20%)	820 (18%)	400 (25%)
M5 (All combinations)	159	300	1402	441
M5 (Average per gene)	0.56	0.48	0.35	0.30
M4 (Single occurrence)	14 (4%)	NE	NE	NE
M4 (All combinations)	132	–	–	–
M4 (Average per gene)	0.60	–	–	–
M6 (Single occurrence)	06 (2%)	NE	NE	NE
M6 (All combinations)	63	–	–	–
M6 (Average per gene)	0.21	–	–	–
M10 (Single occurrence)	12 (4%)	111 (14%)	816 (18%)	NE
M10 (All combinations)	106	264	1442	–
M10 (Average per gene)	0.55	0.60	0.57	–
M11 (Single occurrence)	06 (2%)	035 (04%)	170 (04%)	NE
M11 (All combinations)	93	128	422	–
M11 (Average per gene)	0.28	0.17	0.10	–
No Motif Predicted	17 (5%)	102 (13%)	916 (20%)	617 (39%)
In combinations	232 (69%)	219 (28%)	835 (19%)	041 (3%)
Most frequent combination of motifs				
M2 M5	7% [0.6%]	3% [0.2%]	2% [0.3%]	3% [0.4%]
M10 M5	2% [0.4%]	8% [4%]	7% [1%]	NA
M2 M4	3% [0.3%]	NA	NA	NA
M10 M2	3%	4%	3%	NA
M11 M5	3%	3%	2%	NA
M10 M11	2%	3%	2%	NA
M4 M5 M6	4%	NA	NA	NA
M4 M5	4%	NA	NA	NA

Legend: *NE* not enriched, *NA* not applicable. Values in square brackets were from MAST analysis

Only 5% of genes in VH class lacked any of the predicted motifs. Absence of predicted motifs increased with decreasing expression, and 39% genes of L class had no predicted motifs. Combinations of more than one motif per gene were seen very frequently in high expression class (69%) compared with low expression class (3%). This is in keeping with the association of these motifs with relatively high gene expression. Although motifs were found very frequently in combination with other motifs, no particular combination was highly frequent. The M2 M5 combination was most frequent in VH class, while M10 M5 was most frequent in H and M classes. L class showed only M2 M5 combination, as many of the motifs were not enriched in this class. We checked the motif co-occurrence frequency for the more frequent combinations using MAST with E-value cut-off < 0.05. This analysis showed M10 M5 combination to be significant in high expression class (Table 3).

### Experimental validation of motifs

**Fig. 7:**
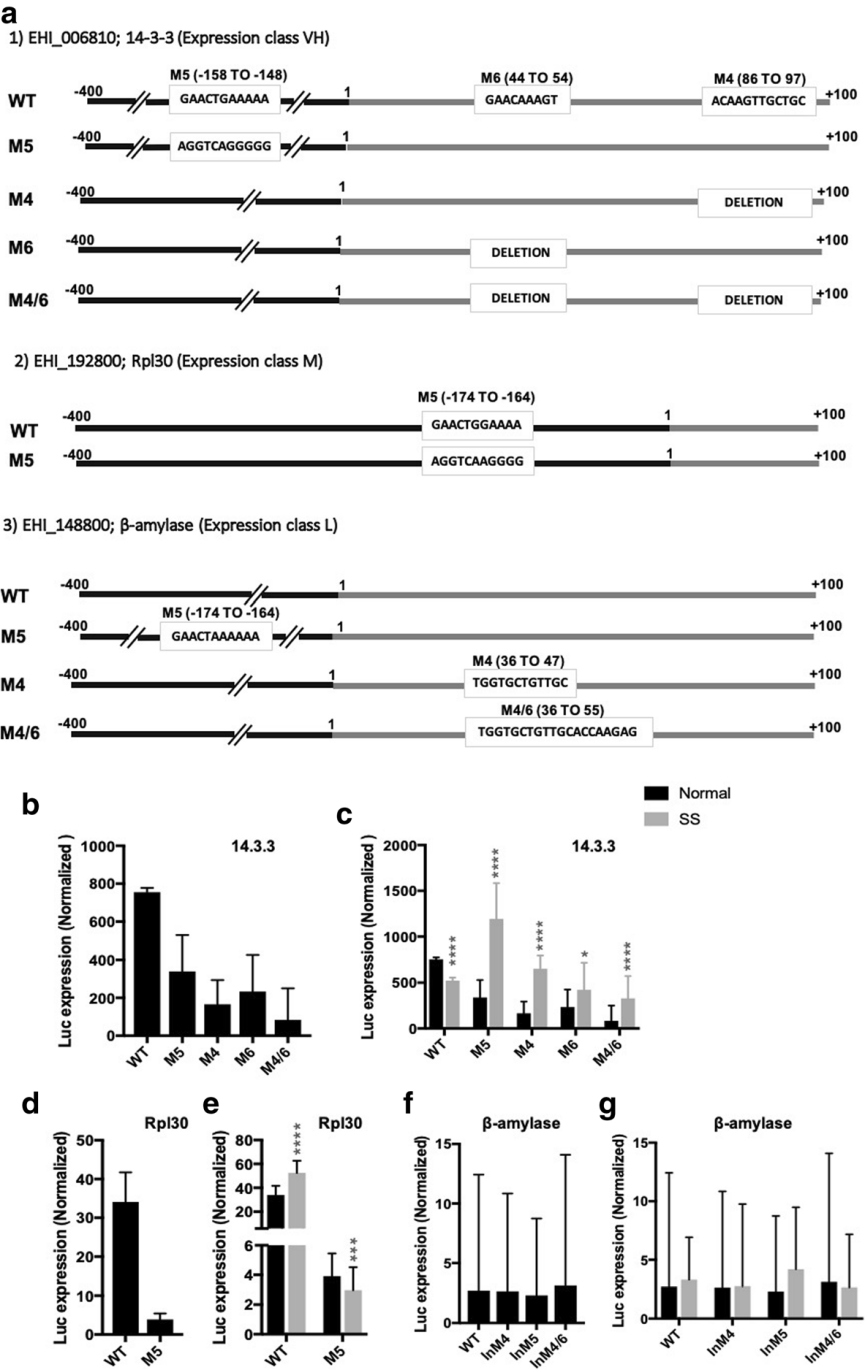
Luciferase expression driven by WT and mutant motifs of selected genes in normal and serum starved (SS) cells. Sequences with the motifs to be tested were cloned upstream of the reporter sequence (Additional file 2). (**a**) The schematic showing sequence changes introduced in each construct with respect to the corresponding motifs in WT sequence are shown for each gene. Luciferase expression as a consequence of mutations in the indicated motifs of selected genes - (**b**) and (**c**): 14.3.3; (**d**) and (**e**): Rpl30; (**f**) and (**g**): β-amylase. In β-amylase, the effect of motif insertion was tested. SS-serum starved

To confirm the functional importance of the predicted motifs in gene expression we selected three genes belonging to different expression classes- 14-3-3 (EHI_006810) very high expression, Rpl30 (EHI_192800) medium expression, and ß-amylase (EHI_148800) low expression class. 14–3-3 contained a number of predicted motifs both upstream and downstream of the AUG start codon. These included motifs M10 (− 380 to − 369), M5 (− 158 to − 148), M2 (− 53 to − 43), M6 (44 to 54), M4 (86 to 97) and M1 (multiple positions). We individually mutated motifs M4, M5 and M6 (which occurred only once in 14–3-3), and also made a double mutant of M4 and M6 (Fig. 7a). Rpl30 contained only a single motif, M5 (− 174 to − 164). Transition mutations were generated for M5 motif in 14–3-3 and Rpl30 gene, while the motifs M4 and M6 of 14–3-3 were deleted keeping the reading frame intact. ß-amylase contained multiple copies of a single motif, M1. In this case we checked whether insertion of motifs from high expression genes could enhance expression. One copy of each of the motifs M4, M5, M6 and M4 + M6 together were inserted at the conserved positions of the respective motif, where they naturally occur most frequently in the data set. Precautions were taken so as not to shift the reading frame. The constructs with WT and mutant motifs were cloned in pEhNeoluc vector of *E. histolytica* (see Additional file 4) upstream of luciferase reporter gene. Stably expressing *E. histolytica* cell lines were obtained by transfection, and the expression of luc was measured. In constructs with WT motifs, 14–3-3 showed the highest expression followed by Rpl30 and ß-amylase, as previously described (Fig. 1). All mutant constructs of 14–3-3 showed decreased expression. Mutations in M4, M5 and M6 caused a decrease in expression of 75, 50 and 66% respectively. The double mutation M4 + M6 showed a drastic decrease of 90%. Rpl30 expression was also drastically reduced by ~ 90% with the mutation in M5 motif (Fig. 7). These data show that the motifs identified by us are, indeed, functionally significant and required for optimal gene expression. Insertion of motifs from high expressing genes into the low expressing ß-amylase gene did not result in significant change in the expression of luciferase (Fig. 7). Thus in the presence of factors responsible for low expression, mere insertion of a motif from high expressing genes appears to be insufficient.

Next, we checked for any change in the expression of these genes during serum starvation. 14–3-3 was slightly down regulated during serum starvation as was also seen in transcriptome data. Interestingly, the drop-in luciferase expression observed by mutating motifs M4 and M5 of 14–3-3 in normal grown cells was not seen in serum starved cells. In fact the expression levels were higher than normal in the mutant constructs. Mutations in motifs M6 and M4 + M6 also had similar (though less pronounced) effects (Fig. 7). The tested motifs appear to be less critical for expression of 14–3-3 in serum starved cells. In the case of Rpl30 and ß-amylase genes there was no significant change during serum starvation and the expression pattern was similar to control cells. (Fig. 7).

### Core promoter motifs in *E. histolytica* genes of all expression classes

Earlier studies have demonstrated three core promoter motifs in *E. histolytica* genes, with consensus sequences- (1) TATA-box (GTATTTAAA) located at − 30, (ii) a GAAC element (AATGAACT) located variably between the TATA and Inr, and (iii) an Inr (AAAAATTCA), overlying the site of transcription initiation [29–31]. The in silico search of these motifs has been reported in a limited set of 246 *E. histolytica* genes [32]. We searched for these motifs in our entire set of annotated genes (*n* = 7597) covering all expression classes. (see Additional file 14). Since all three motifs were localized within 60 bp upstream to the site of transcription initiation, we extracted 100 nt upstream of AUG to scan for the core promoter motifs. FIMO was used for motif scanning [33] with default *p*-value (1E-4). At least one of the three core promoter motifs could be identified in 1478 of the 7597 genes (19.45%) (Table 4). We identified 589 genes with consensus TATA box sequences, 734 genes with GAAC motif and 90 genes with Inr motif. Only 46 genes had both TATA and GAAC box, 6 genes had both TATA and Inr motifs and 13 genes had both GAAC and Inr motifs. We could not find any sequence having all the three motifs together. Further, core promoter motifs were found in genes of all expression classes, and there was no preferential association with any expression class (Table 4). We also checked the location of core promoter motifs. TATA box was located predominantly at − 50 to − 30, and GAAC box peaked at − 40 to − 20. This is in agreement with previous studies [29]. The Inr motif, however, was found only in a small number of genes and did not appear enriched at any specific location (see Additional file 15 a, b).

**Table 4:** *In silico* identification of core promoter elements in genes of each expression class

Core promoter elements	Expression Class					
	VH	H	M	L	VL	Total genes
Genes with core promoter motifs/total genes	84/338 (25%)	217/778 (28%)	810/4475 (18%)	280/1597 (18%)	87/409 (22%)	1478/7597 (20%)
TATA box	40	84	257	137	71	589
GAAC box	37	102	462	120	13	734
Inr box	3	15	57	13	2	90
Genes with TATA and GAAC	4	13	22	7	0	46
Genes with TATA and Inr	0	2	3	1	0	6
Genes with GAAC and Inr	0	1	9	2	1	13
Genes with All three	0	0	0	0	0	0

### Core promoter motifs in genes containing downstream motifs (M4 and M6)

The downstream motifs M4 and M6 were uniquely present only in the VH expression class. We checked the status of core promoter elements in genes with M4 and M6 motifs since it has been seen that DPEs, first reported in Drosophila, are more commonly associated with TATA-less promoters [27, 34]. To check if downstream motifs in *E. histolytica* were also associated with lack of upstream core promoter motifs, we scanned all genes with M4 and/or M6 motifs (*n* = 160) for the presence and location of core promoter motifs as described above. We identified 19 genes with consensus TATA box sequences, 18 genes with GAAC motif, 3 genes with both TATA and GAAC motifs, and no genes with Inr motif. The core promoter motif frequency and location in these genes was similar to that observed for all *E. histolytica* genes (see Additional file 15c, d). Thus, in *E. histolytica*, the downstream motifs M4 and M6 are unlikely to be linked with TATA-less promoters. Their association with highly transcribed genes may be mechanistically different from the core DPE motifs of other organisms.

### Expression status and predicted motifs of cysteine proteases (CPs) and other virulence-related genes

It was interesting that many of the virulence-related genes were highly transcribed in axenic culture. CPs are implicated in amoebic virulence and have been extensively studied in *E. histolytica*. We analysed their expression pattern and compared with the previous studies. Of the 35 papain-like *E. histolytica* CP genes [35, 36] we identified 28 in our RNA-Seq data. Their expression ranged from very low to very high. Comparison of our expression data with that of Tillack et al. [35], showed very good overall concurrence (see Additional file 16). Amongst the highly expressed CP genes in our data, only one (EHI_010850) did not appear in earlier studies (see Additional file 16). Our data helped to refine the expression-based categorization of EhCP genes. The bulk of EhCP transcription was contributed by three genes- EhCP-A2, A1 and A5 (in VH class). Although their expression was down regulated during serum starvation, they were still the most highly transcribed. However, high transcript levels are not necessarily correlated with high CP activity as shown in a comparison between pathogenic and non-pathogenic *E. histolytica* clones, and regulation of CP enzyme levels is complex [36].

We looked for the predicted promoter motifs in CP genes. Of the 28 genes we could identify at least one motif in 21 genes, some of which had more than one motif, bringing the total number of motifs to 25 (ignoring the multiple occurrences of the same motif). Motifs M1 and M10 were most commonly found (15/25), especially in the high expression class. Motif M5 was also common (6/25). Upon serum starvation three of the CP genes were significantly down regulated (at least 2-fold), while only one was up regulated. Interestingly, the down regulated genes belonged to high expression class, while the up regulated gene was in low expression class. Increased expression of normally low-expressing CP genes was also found in trophozoites from amoebic liver abscess [36].

The distribution of other virulence-related genes in various expression classes, and commonly occurring motifs was also determined (Table 5). Data showed that most virulence-related genes were expressed at moderate to high levels, with only AIG1 family showing low expression. The most common motifs were M10, M5 and M1. Motifs M4 and/or M6 were found in two of the amoebapores, and in grainin genes. All four amoebapore isoforms were highly expressed. Although the constitutive expression of amoebapores has been reported in axenically grown *E. histolytica* [37, 38], their high expression is intriguing as their known function is to lyse bacteria or other surrounding cells by membrane insertion following contact of target cell with the trophozoite. This cytotoxic activity would not be required in axenically grown cells, and possibly indicates that these pore forming peptides might have other constitutive functions.

**Table 5:** Expression status and motif occurrence in virulence-related genes

Gene	No. of members	Expression class					Most common motifs
		VH	H	M	L	VL	
Amoebapore	4	4	–	–	–	–	M10 > M4 = M6 = M1
CP	28	3	3	13	6	3	M10 > M1 > M5 > L_M3 = L_M4 = M11
C2 domain containing	10	1	2	5	2	–	M5 = M1 > M10 > M11
©AIG1	28	–	–	8	15	5	M5 > M10 = M1 = M2 = L_M4
Gal/GalNac lectin	14	3	8	3	–	–	M1 > M5 > M10 > M2 = M11
Grainin	4	2	2	–	–	–	M4 = M5 > M1
Lysozyme	4	1	–	3	–	–	M5 > M1 > M10
Myb family	24	–	2	18	3	1	M1 > M10 = M2 > M5 > M11
Peroxiredoxin	10	6	2	1	1	–	M11 > M10 > L_M3
20 kDa antigen	2	1	1	–	–	–	M1 > M5
Surface antigen ariel1	10	4	2	1	1	2	L_M4 > L_M3 = L_M9 = M1

Legend: Multiple occurrence of a motif in the same gene was not considered.©Several members of these genes had no predicted motif

### Transcriptomic changes during serum starvation

We used serum starvation to induce nutritional stress due to which cell division stopped but cell viability was maintained for at least 24 h. Differential gene expression analysis between normal and serum starved cells was performed using cuffdiff program of cufflinks package with default settings (at *P*-value <= 0.05). Two-fold change (Log2 FC > =1) was set as a cut-off for differential expression. We found 185 genes up regulated in serum starved cells as compared to normal, while 157 were downregulated (see Additional files 17 and 18). Of the 185 up regulated genes, 93 were hypothetical and were not considered. Genes showing the maximum up regulation (Log_2_ FC > = 2) mostly belonged to the low or medium expression class. GO analysis of these genes is given in Table 6. They included genes involved in signalling pathways (kinases and GTPases), lipid metabolism, DNA repair factors, translation factors, Myb family of transcription factors, BspA family proteins, heat shock proteins and cell cycle regulators.

**Table 6:** GO analysis of differentially expressed genes

Condition		Gene No.	Category	Term	Count	%	*P*-value	Enrichment
Up regulated	N vs. SS	185 (92)	BP	GO:0044699~single-organism process	35	39.8	0.006	1.40
			CC	GO:0005622~intracellular	39	44.3	0.005	1.28
			MF	GO:0016740~transferase activity	24	27.3	0.05	1.46
	SS vs. SR	32 (16)	MF	GO:0003824~catalytic activity	13	81.25	5.59E-04	1.86
Down regulated	N vs. SS	157 (109)	BP	GO:0008152~metabolic process	47	43.9	0.006	1.29
			MF	GO:0003824~catalytic activity	64	59.8	1.99E-05	1.42
	SS vs. SR	28 (19)	BP	GO:0006629~lipid metabolic process	4	21.05	0.0036	10.36

Legend: (N: normal growth; SS: serum starved; SR: serum replenishment)

Of the 157 down regulated genes, 48 were hypothetical and were not considered. Genes showing the maximum down regulation (fold change >Log_2_ = 2) mostly belonged to the high or medium expression class. They included genes for signaling factors (Rab/Ras/Rho) GTPase and kinases, actin and actin binding proteins, metabolic pathways, asparagine-rich surface antigen Ariel family proteins, ribosome biogenesis factors, and some cysteine proteases. The down regulation of metabolism related transcripts and actin binding proteins is in keeping with reduced energy requirement and cell motility during serum starvation.

We also looked at up- or down-regulated genes in 24-h serum-starved cells replenished with serum for 2 h. 32 genes were up regulated of which 16 were hypothetical. Some of the genes down regulated in starved cells, particularly surface antigen Ariel1 were up regulated upon serum replenishment. 28 genes were down regulated in serum replenished cells. Of these, 9 were hypothetical. Hsps were amongst the prominent down regulated genes in serum replenished cells recovering from stress (see Additional files 19 and 20).

## Discussion

The release of *E. histolytica* genomic sequence in 2005 [39] led to a series of genome wide transcriptome studies on gene expression profiling in *E. histolytica* [10, 12, 13] and stage-specific gene expression in *Entamoeba invadens* [8, 11, 40]. Previous studies using transcriptome analysis to study regulation of gene expression in *E. histolytica* had defined goals. The study by Hon et al. [13] used RNA-Seq data to assess extent of alternative splicing and polyadenylation. The study by Hackney et al. [12] used microarray data to identify genome-wide transcriptional regulatory patterns. Our study mined RNA-Seq data to analyse genes belonging to different expression classes, and to look for regulatory motifs both upstream and downstream of the AUG start codon, which may be associated with high gene expression.

We show that conserved sequence motifs exist both upstream and downstream of AUG, either alone or in combination, and are required for efficient transcription. Seven such motifs were associated with highly transcribed genes. Specific motifs were associated more frequently with genes of particular functional categories. Motifs M10, M1, M5 and M4 were most commonly seen in virulence-related genes. Two novel downstream motifs (M4 and M6), required for gene expression, were found in some of the very highly expressed *E. histolytica* genes. We tested the functional significance of these motifs in selected genes. The 14–3-3 gene contained a number of predicted motifs both upstream and downstream of AUG, of which we tested three (M4, M5 and M6). Mutations in each of them alone resulted in reduced reporter gene expression and mutating two of these together had an additive effect. These motifs are likely to fine tune the levels of *E. histolytica* gene expression and may have evolved to optimize expression of individual genes. This is also borne out from the behaviour of 14–3-3 motif mutants in serum starved cells, where (unlike in normally growing cells) the mutated motifs did not result in reduced gene expression. It is possible that protein factors that bind to these motifs may be over-expressed in serum starved cells, or a negative regulator may be down regulated. The important transcription regulatory roles of these motifs need to be explored further. Our analysis also showed that the 14–3-3 promoter could be used for enhanced expression of cloned genes in *E. histolytica* as it gave 6-fold higher luciferase reporter gene expression than the lectin promoter commonly used for expressing cloned *E. histolytica* genes in the vector pEhNeoLuc.

Another significant finding of our study was that some of the virulence-related genes like amoebapores and CPs showed high expression in the RNA-Seq data. These genes are possibly required for optimal *E. histolytica* growth, and their functions are additionally utilized for pathogenesis. Our data do not imply that high expression is a prerequisite for virulence as some virulence-related genes, for example those belonging to AIG1 family belonged to medium and low expression class.

Our study also shows that serum starvation can be used to look at transcriptomic changes in gene expression programme induced by nutritional stress. Serum starvation has been shown to stop cell division but cell viability was maintained for at least 24 h. It has been used to generate synchronized cell population in *E. histolytica* [41]. We have earlier used serum starvation to study the regulation of a variety of genes in *E. histolytica*. These included ribosomal-RNA and protein genes where we showed post-transcriptional regulation of rRNAs and r-proteins [42, 43], serum-dependent selective expression of some members of trans membrane kinases (TMKs) [18, 44], a serum-responsive long noncoding RNA [45], and nuclear loss of the exoribonuclease Rrp6 in serum-starved cells [46]. Here we have looked at global regulation of genes in serum-starved cells. We found up regulation of signalling pathway transcripts (kinases and GTPases), which is also reported in different virulence related studies [3, 47]. Myb family transcription factors were also up regulated. These are likely to regulate several different pathways as judged from the presence of putative Myb recognition sequence in the promoters of *E. histolytica* genes [32]. These genes were also up regulated during trophozoite to cyst conversion [8]. BspA family genes were also up regulated in serum-starved cells. Proteins of the BspA family have been located on the *E. histolytica* surface and shown to function in binding to tumor necrosis factor, a chemo attractant required for tissue invasion by *E. histolytica* [48]. It will be interesting to study the common regulatory pathways used by *E. histolytica* to adapt to a variety of stresses, and during stage conversion.

## Conclusions

Transcriptomic analysis of *E. histolytica* revealed that apart from the genes expected to be highly expressed, namely those coding for translation-related, oxidative stress, and cytoskeletal functions; most of the virulence-related genes like amoebapores and CPs were also highly expressed under axenic culture conditions. These genes are possibly required for optimal *E. histolytica* growth, and are additionally utilized for pathogenesis. Two novel downstream motifs, required for gene expression, were found in some of the very highly expressed *E. histolytica* genes. To our knowledge, such downstream motifs have not been reported genome-wide in other protozoan parasites. Their functional analysis may give interesting new insights into control of gene expression.

## Methods

### Cell culture and growth conditions

Trophozoites of *E. histolytica* strain HM-1:IMSS were axenically maintained in TYI-S-33 medium supplemented with 15% adult bovine serum (Biological industries, Israel), Diamond’s Vitamin mix, Tween 80 solution (Sigma–Aldrich) and antibiotics (0.3 units/ml penicillin and 0.25 mg/ml streptomycin) at 35.5°C (Diamond et al., 1978). For serum starvation, medium from early to mid-log phase grown trophozoites (48 h) was replaced with TYI-S-33 medium containing 0.5% adult bovine serum and incubation continued for 24 h. Replenishment was achieved by decanting total medium and replacing with complete TYI-S-33 medium. G-418 (Sigma) was added at 10 μg/ml for maintaining the transfected cell lines.

### RNA isolation and transcriptome analysis

Cells were harvested from normal growth, or after 24 h of serum starvation, or after 2 h of serum replenishment. Total RNA from ∼5 × 10^6^ cells was purified using TRIzol reagent (Invitrogen) according to the manufacturer’s instructions.

Total RNA, from two biological replicates for each of the three samples, was used for selection of polyA plus RNA and library preparation was done after oligo (dT) selection. RNA-Seq libraries were generated by performing RNA fragmentation, random hexamer primed cDNA synthesis, linker ligation and PCR enrichment. These libraries were then subjected to paired-end sequencing on the Illumina HiSeq2500 (v3 Chemistry) platform. A total of 42,64,58,460 RNA reads of size 100 bp were generated from the 6 samples. After trimming adapter sequences and removing low quality reads using trimmomatic-0.36, we got 6.4 to 7.6 million reads per sample [49]. The GC content was 33% and the percentage of reads with ≥ Q30 were 99.95% in all three samples. On an average, ~ 92.70% of total high quality reads (39,53,69,451) aligned to the reference sequence. The pre-processed reads were aligned to the *E. histolytica* (HM1:IMSS) genome for which the gene model was downloaded from AmoebaDB (http://amoebadb.org/common/downloads/release-27/EhistolyticaHM1IMSS/gff/data/). The alignment was performed using Tophat program (version 2.0.11) with default parameters. To evaluate the quality of RNA-Seq data from features like read distribution, 5′/3′ bias and sequencing depth we used RSeQC tool (version 3.0.0) [50]. The bam files from all the six samples were merged for RSeQC analysis. The bed file used for this analysis was generated from the *E. histolytica* gff file, downloaded from AmoebaDB, release 27 (http://amoebadb.org/common/downloads/release-27/EhistolyticaHM1IMSS/gff/data/). The RSEM program (version 1.3.0) was used for estimating expression of the genes and transcripts [51]. The differential gene expression analysis was performed using cuffdiff program of cufflinks package with default settings to analyse the difference between normal and serum starved cells. Log_2_ fold change was set as a cut-off for differential expression.

### Real time qRT PCR

2 μg of total RNA (DNase I treated) was reverse transcribed using random hexamers by Verso reverse transcriptase (Invitrogen) in a reaction volume of 20ul. Real time quantitative PCR was performed in 7500 Real Time PCR System (Applied Biosystems) using SYBR green PCR Master Mix, 2 pmol of forward and reverse primers and 2 μl of cDNA. For transcriptome data validation and quantitative comparison 18S rRNA (control gene, 1:100 dilution) and a few other genes including GAPDH, Actin and TMKB1–18 and those belonging to different classes of expression were amplified in parallel. The conditions were pre-denaturation at 95 °C for 10 min, followed by 40 cycles at 95 °C for 15 s and 58 °C for 1 min followed by a dissociation stage at 95 °C for 15 s and 58 °C for 1 min. Cycle threshold values (Ct) were analysed by the SDS1.4 software (Applied Biosystems) and all samples were analysed in triplicate, in three independent experiments. Reactions without cDNA were used as no template control (NTC) and -RT controls were set up to rule out genomic DNA contamination. The relative expression with respect to serum starvation was plotted and compared to the RNA Seq data.

### In silico regulatory motifs prediction

In silico prediction of regulatory motifs were elucidated by using the command line mode (version 4.12.0) of MEME in discriminative mode [52]. It requires a background file, position-specific priors (PSP) file, training set and test set. To make the motif search robust, providing an appropriate background model is one of the most important factors. For that we generated random sequences of the same nucleotide composition as *E. histolytica* using “random DNA sequence generator” [53], which were used to generate a 2nd order background model using fasta-get-markov*.* This background model was used at each step of the motif search process. For discriminative search, PSP were calculated from negative control sequences and training sequences using PSP-gen [23]. The PSP file is used in addition to the background model to bias the search for motifs at specific positions in the input sequences. Sub-sequences that appear in both the primary sequence and the control sequence sets are considered less likely to be instances of motifs. MEME was run in discriminative mode using the command line: -dna -bfile -psp -mod zoops -minw 6 -maxw 12 -nmotifs 20 -maxsize 6,000,000. This predicted 20 motifs of width range from 6 to 12.

In order to predict the regulatory motifs enriched in genes with different levels of gene expression we proceeded by making training set of very high expression genes and used very low expressing genes as a negative control. We split the very high expressing genes (*n* = 343) into a training and test set by allocating 2/3 (*n* = 228) of genes for training. We repeated the process thrice by randomly selecting genes for training and test set. To make the search more robust, we also generated another training set by allocating 1/2 (*n* = 171) of genes and again repeated the process thrice by randomly selecting genes for training set. Thus, for prediction of motifs belonging to each class (very high in this case) six round of random selections were made. The motifs which appeared in more than four searches showed very high probability.

The predicted motifs from the training sets were analysed for their enrichment in test sets i.e. the remaining 1/3 (*n* = 114) and 1/2 (n = 171) of very high expressing gene class. Moreover, to distinguish the motifs among different class of expression, the predicted very high expression class motifs were examined for enrichment in gene dataset of high, medium and low classes by keeping the very low gene expressing class as a negative control. For motif enrichment analysis we used AME [23] with the same background model and negative control. AME tool of the MEME suite provides motifs that are enriched compared to the shuffled background. Motifs with adjusted enrichment *p*-value ≤0.01 and occurrence in a minimum of four rounds of six rounds for a specific expression class were considered enriched. These enriched motifs were further subjected for FIMO [33] search to locate individual motif occurrence in the expression class they were predicted for.

The same steps were followed to predict the regulatory motifs which could possibly correlate with the low level of gene expression. Very low expressing genes (*n* = 469) were used to generate training set (2/3 and 1/2) while very high expressing genes were set as a negative control.

For the functional annotation of genes associated with predicted motifs, Gene Ontology (GO) terms were generated by DAVID Bioinformatics Resources (version 6.8). Enriched GO terms were characterized by biological process (BP), molecular function (MF) and cellular component (CC). The threshold EASE scores (modified Fisher exact *P*-value) for gene-enrichment analysis was set as < 0.01 for BP, MF and CC.

### Luciferase reporter constructs and stable transfection

The 500 bp sequence spanning -400 bp to + 100 bp wrt AUG was extracted for the selected genes (14–3-3, RPL30 and ß-amylase) belonging to different expression classes. Sequences were amplified from genomic DNA of *E. histolytica* by PCR using indicated primers (see Additional file 21). To modify the motif sequence, overlapping PCR was performed. Constructs were gel cut purified and cloned upstream of LUC gene at XhoI/Acc651 site in pEh-NeoLuc vector (see Additional file 4). Sequencing of the constructs was done to confirm the modification. Stable transfection was done by electroporation as described previously [54]. Briefly, trophozoites in log phase were harvested and washed with phosphate-buffered saline (PBS) followed by incomplete cytomix buffer (10 mM K_2_HPO^4^/KH_2_PO^4^ (pH 7.6), 120 mM KCl, 0.15 mM CaCl_2_, 25 mM HEPES (pH 7.4), 2 mM EGTA and 5 mM MgCl_2_). The washed cells were then re-suspended in 0.8 ml of complete cytomix buffer (incomplete cytomix containing 4 mM ATP and 10 mM glutathione) containing 200 μg of plasmid DNA and subjected to two consecutive pulses of 3000 V/cm (1.2 kV) at 25 mF (Bio-Rad, electroporator). The transfectants were initially allowed to grow without any selection. Drug selection was initiated after 2 days of transfection in the presence of 10 μg/ml G-418. After the cells were stable Luciferase assay was performed.

### Luciferase assay

This was done as described previously [55]. Briefly, stably transfected trophozoites, maintained in TYI-S-33 medium supplemented with 10 μg/ml G-418, were chilled on ice, harvested and washed twice in 1XPBS (pH 7.4) and lysed in200ul of 5X RLB (reporter lysis buffer, Promega) with the addition of protease inhibitors E64-C, leupeptin and nuclease free water to make the final concentration of 1X. Lysates were frozen at − 80 °C. After thawing on ice for 10 min, lysate was centrifuged to remove cell debris and the samples were allowed to warm to room temperature. Luciferase activity was measured according to the manufacturer’s instructions (Promega) using a Turner Luminometer (model TD-20E). Luciferase activity per microgram of protein was calculated as a measure of reporter gene expression. The luciferase activity was done in triplicate for each sample. The protein concentration of the lysates was measured by the BCA method using a solution of CuSo4: BCA (1:50), and BSA standard.

## Additional files

Additional file 1: Read alignment summary. (DOCX 14 kb)

Additional file 2: a) Distribution of mapped reads over genome features like exon, intron and intergenic region; b) Coverage uniformity over gene body. All transcripts were scaled into 100 nt.; c) Saturation analysis of splice junction detection. Annotation of detected (d) splice junctions, and (e) splice events. ‘known’: splice junctions with both 5′ splice site (5′ SS) and 3′ splice site (3′ SS) annotated by reference gene model; ‘complete novel’: splice junctions with neither 5′ SS nor 3′ SS annotated by reference gene model; ‘partial novel’: splice junctions with either 5′ SS or 3′ SS annotated by reference gene model. (JPG 159 kb)

Additional file 3: Correlation coefficient between the duplicate samples of Normal, Serum Starved and Serum Replenished. (JPG 110 kb)

Additional file 4: p*Eh*NeoLuc Vector Map. (JPG 32 kb)

Additional file 5: GO analysis of genes with different motifs and expression class. (XLSX 88 kb)

Additional file 6: List of genes belonging to very high class. (DOCX 21 kb)

Additional file 7: List of genes belonging to very low class. (DOCX 23 kb)

Additional file 8: Motifs in Eh. (XLSX 748 kb)

Additional file 9: Positions of motifs M1, M2 and M5 with respect to AUG (A = 1). (JPG 369 kb)

Additional file 10: Positions of motifs M4 and M6 with respect to AUG (A = 1). The position of the motifs present in negative control is also plotted. (JPG 88 kb)

Additional file 11: Localization of motifs M4 and M6. The location of M4 and M6 is shown in sequences flanking AUG (position 1) in all genes containing these motifs. Their overlap was checked by MAST. Eight sequences showed overlap of motif M4 and M6 with significant *p*-value (≤0.01); all 8 sequences belong to actin gene (right panel). (JPG 86 kb)

Additional file 12: Positions of motifs M10 and M11 with respect to AUG (A = 1). (JPG 208 kb)

Additional file 13: Positions of motifs L_M3, L_M4 and L_M9 with respect to AUG (A = 1). (JPG 212 kb)

Additional file 14: Core promoter sequence in Eh genes. (XLSX 75 kb)

Additional file 15: In silico identification of core promoter elements in *E. histolytica* promoters. (a) Venn diagram displaying the total number for genes having each element and their co-occurrence. (b) Positions of core promoter motifs. No sharp peak was seen for Inr motif owing to its comparatively low number (*p* value: 0.0001). (c) Core promoter motifs in genes with downstream motifs (M4 and M6). (d) conserved position in these genes (p-value: 0.0001). (JPG 107 kb)

Additional file 16: Status of cysteine protease expression in comparison to Tillack et al., 2007 and Matthiesen et al., 2013 [35,36]. (DOCX 20 kb)

Additional file 17: Up regulated genes in serum starved cells. (Of the 185 up regulated genes 93 were uncharacterized). (DOCX 23 kb)

Additional file 18: Down regulated genes in serum starved cells (Of the 157 down regulated genes 48 were uncharacterized). (DOCX 25 kb)

Additional file 19: Up regulated genes during serum replenishment (Of the 32 up regulated genes 16 were uncharacterized). (DOCX 15 kb)

Additional file 20: Down regulated genes during serum replenishment (Of the 28 down regulated genes 09 were uncharacterized). (DOCX 16 kb)

Additional file 21: Primer List. (DOCX 16 kb)

## Abbreviations

AME: Analysis for motif enrichment
CP: Cysteine protease
DPE: Downstream promoter element
FAC: Functional annotation clustering
FIMO: Find Individual motif occurrences
GO: Gene ontology
H: High
L: low
M: Medium
MAST: Motif alignment and search tool
MEME: Multiple EM for Motif Elicitation
N: Normal
PSP: Position specific prion
RSEM: RNA-Seq by expectation-maximization
SR: Serum replenishment
SS: Serum starved
TPM: Transcripts per million
VH: Very high
VL: Very low
