# Supplementary material for: Transcriptomic analysis reveals novel downstream regulatory motifs and highly transcribed virulence factor genes of Entamoeba histolytica
Source: BMC Genomics. 2019 Mar 12;20:206. doi: 10.1186/s12864-019-5570-z (PMC6416950; doi:10.1186/s12864-019-5570-z)
Supplement: Supplementary file 1 — Read alignment summary. (DOCX 14 kb) [file 12864_2019_5570_MOESM1_ESM.docx]

**Additional file 1:** Read alignment summary

| **Sample** | **Reads** | **QC Passed** | **QC Passed %** | **Aligned Read Count** | **Aligned %** |
| --- | --- | --- | --- | --- | --- |
| N(1) | 7,16,19,108 | 7,15,82,096 | 99.95% | 6,74,77,467 | 94.27% |
| SS(1) | 7,40,72,322 | 7,40,31,056 | 99.94% | 6,95,58,752 | 93.96% |
| SR(1) | 7,18,91,334 | 7,18,49,166 | 99.94% | 6,81,72,364 | 94.88% |
| N(2) | 7,69,86,582 | 7,69,63,844 | 99.97% | 7,08,18,568 | 92.02% |
| SS(2) | 6,46,01,612 | 6,45,77,156 | 99.96% | 5,84,80,174 | 90.56% |
| SR(2) | 6,72,87,502 | 6,72,70,406 | 99.97% | 6,08,62,126 | 90.47% |

N, normal; SS, serum starved; SR, serum replenished after starvation
