# Supplementary figures and images for: Transcriptomic analysis reveals novel downstream regulatory motifs and highly transcribed virulence factor genes of Entamoeba histolytica
Source: BMC Genomics. 2019 Mar 12;20:206. doi: 10.1186/s12864-019-5570-z (PMC6416950; doi:10.1186/s12864-019-5570-z)

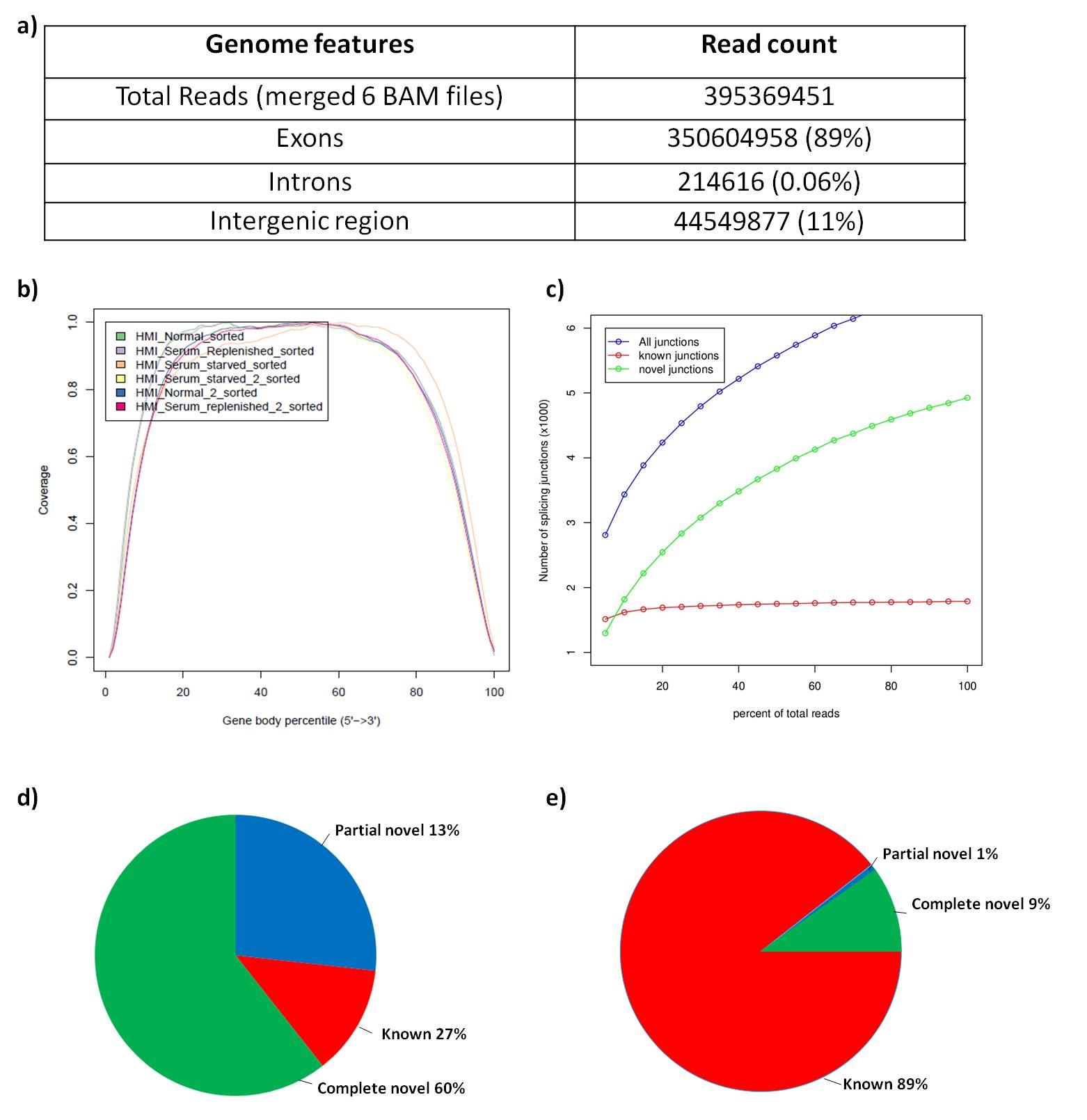

Supplement: Supplementary file 2 — a) Distribution of mapped reads over genome features like exon, intron and intergenic region; b) Coverage uniformity over gene body. All transcripts were scaled into 100 nt.; c) Saturation analysis of splice junction detection. Annotation of detected (d) splice junctions, and (e) splice events. ‘known’: splice junctions with both 5′ splice site (5′ SS) and 3′ splice site (3′ SS) annotated by reference gene model; ‘complete novel’: splice junctions with neither 5′ SS nor 3′ SS annotated by reference gene model; ‘partial novel’: splice junctions with either 5′ SS or 3′ SS annotated by reference gene model. (JPG 159 kb) [file 12864_2019_5570_MOESM2_ESM.jpg]

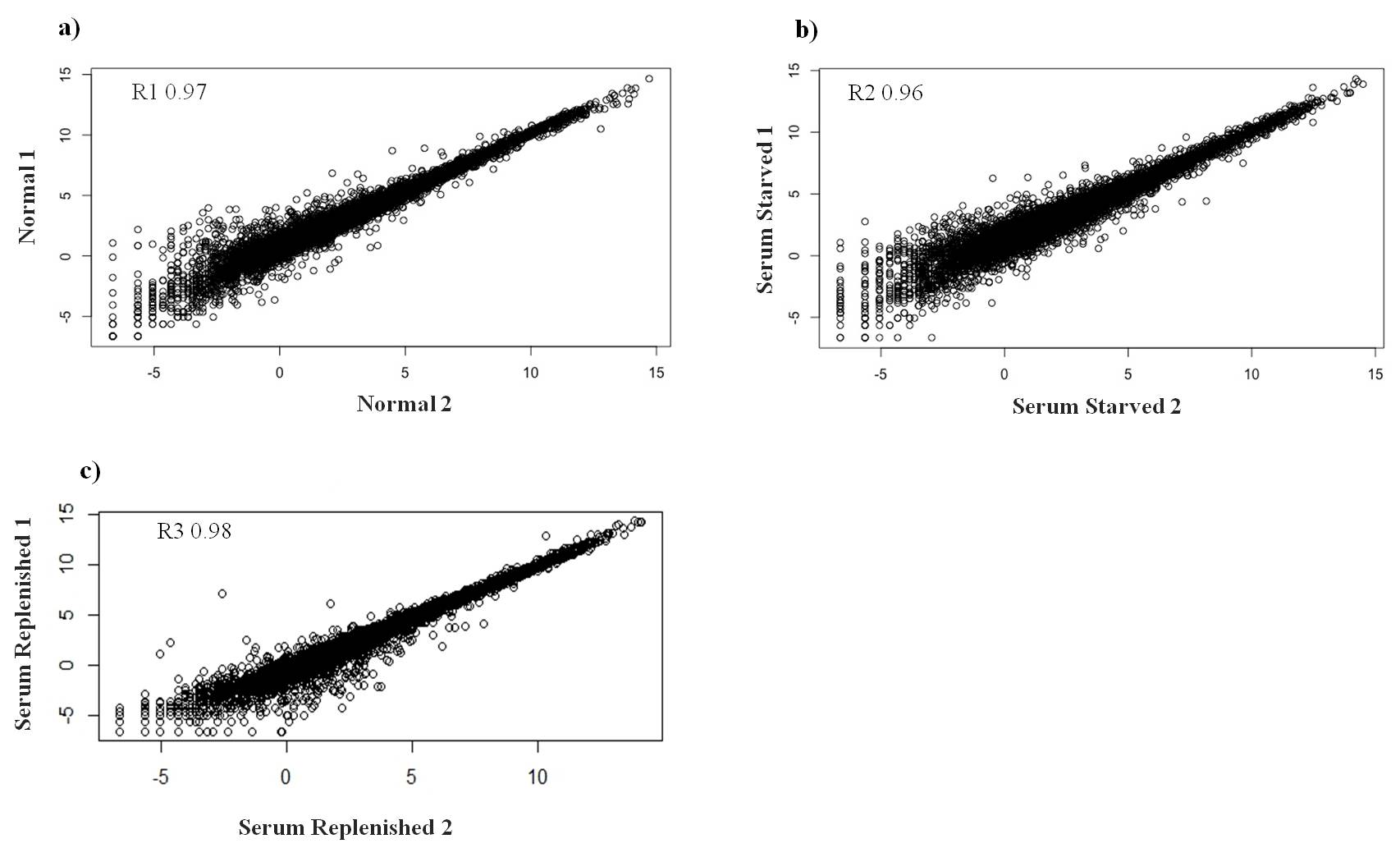

Supplement: Supplementary file 3 — Correlation coefficient between the duplicate samples of Normal, Serum Starved and Serum Replenished. (JPG 110 kb) [file 12864_2019_5570_MOESM3_ESM.jpg]

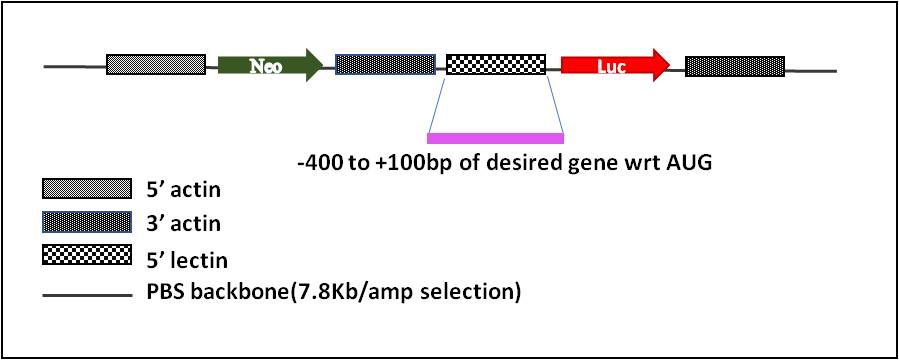

Supplement: Supplementary file 4 — pEhNeoLuc Vector Map. (JPG 32 kb) [file 12864_2019_5570_MOESM4_ESM.jpg]

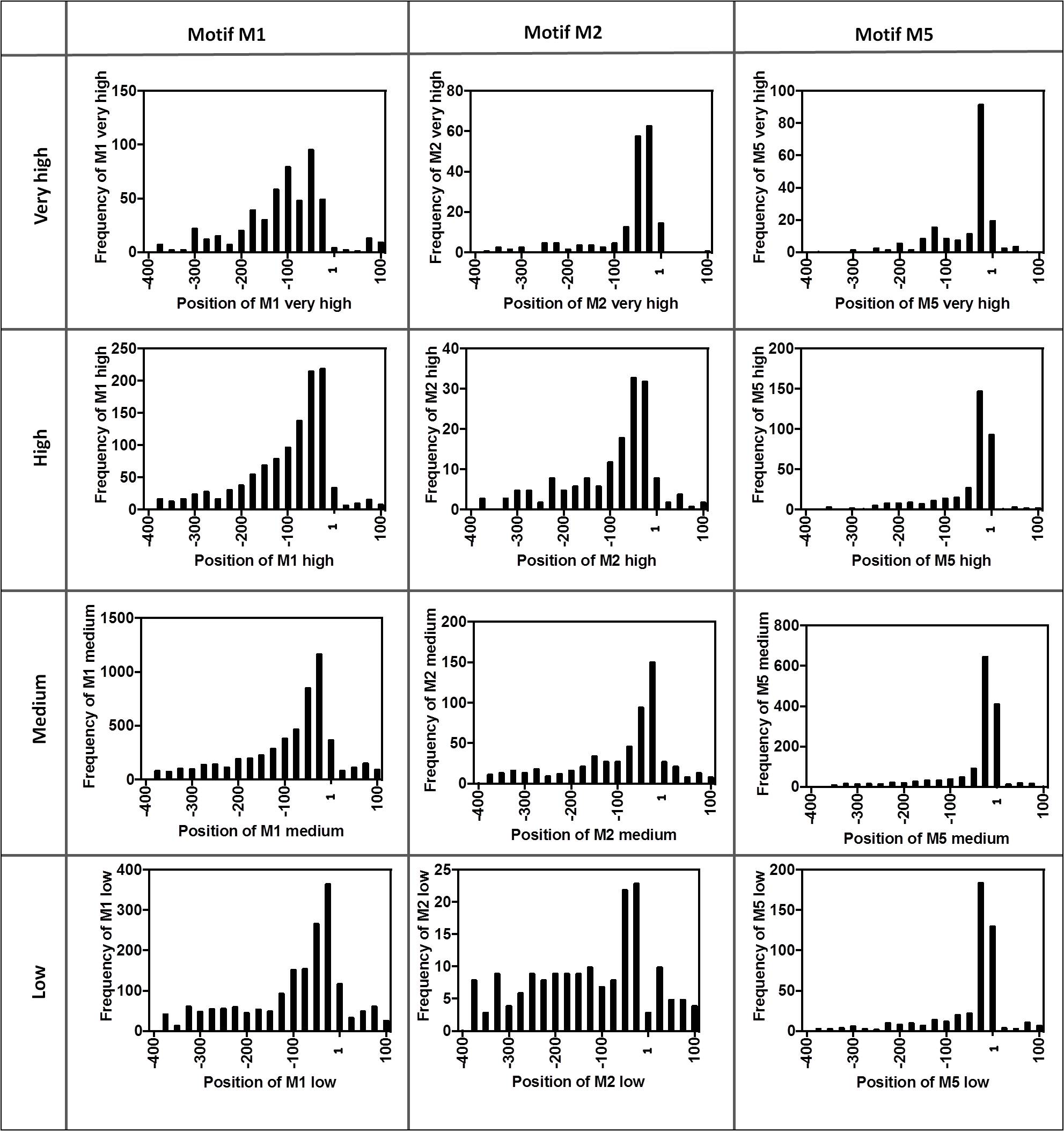

Supplement: Supplementary file 9 — Positions of motifs M1, M2 and M5 with respect to AUG (A = 1). (JPG 369 kb) [file 12864_2019_5570_MOESM9_ESM.jpg]

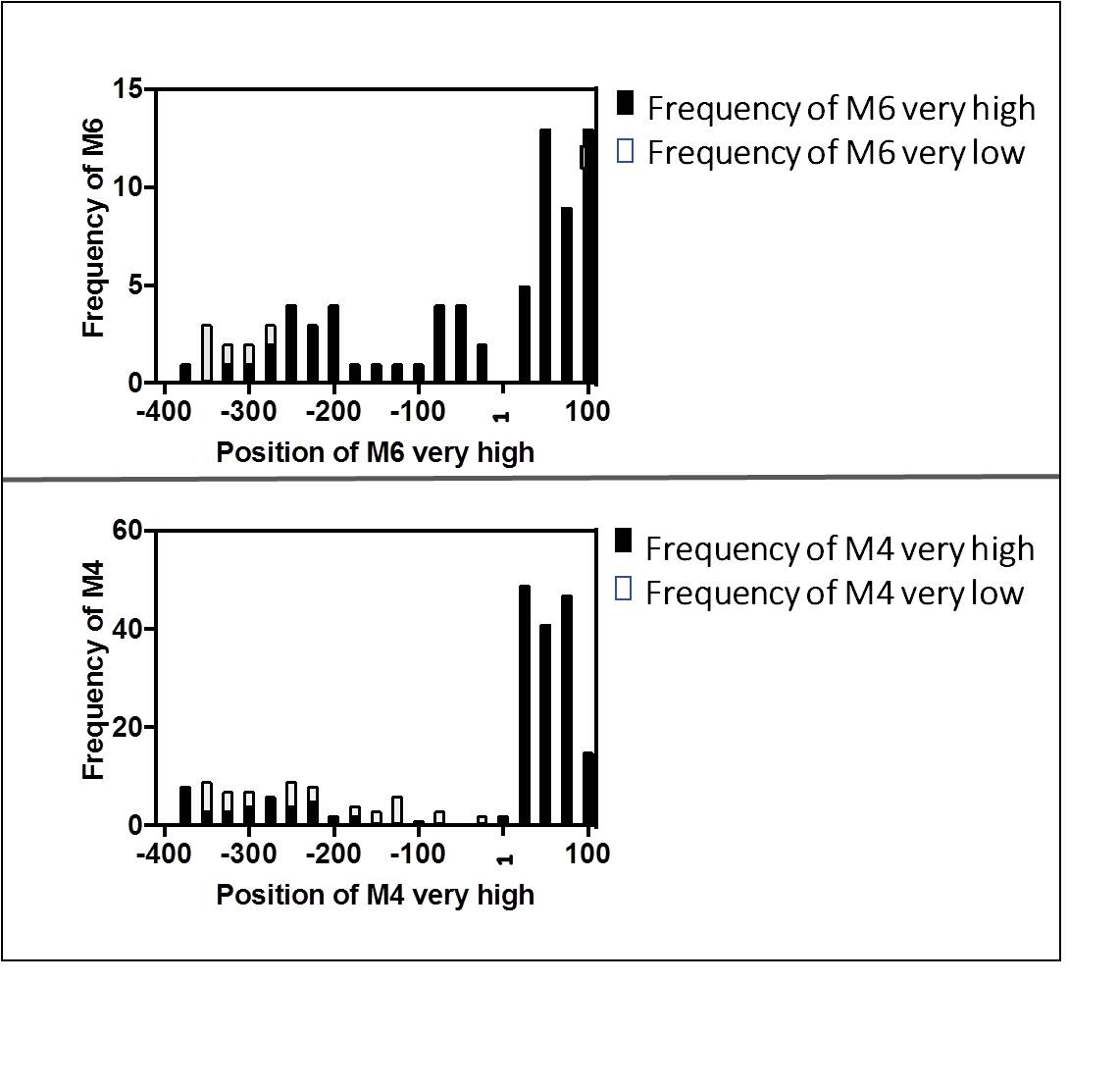

Supplement: Supplementary file 10 — Positions of motifs M4 and M6 with respect to AUG (A = 1). The position of the motifs present in negative control is also plotted. (JPG 88 kb) [file 12864_2019_5570_MOESM10_ESM.jpg]

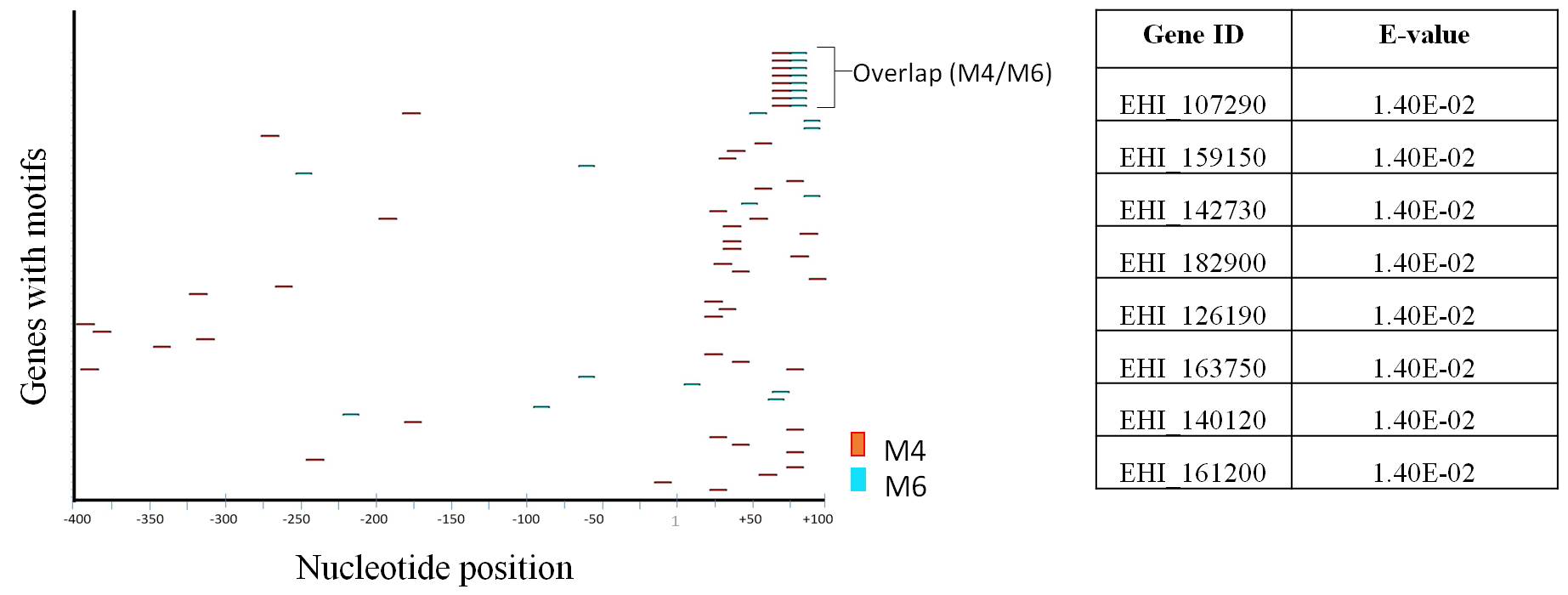

Supplement: Supplementary file 11 — Localization of motifs M4 and M6. The location of M4 and M6 is shown in sequences flanking AUG (position 1) in all genes containing these motifs. Their overlap was checked by MAST. Eight sequences showed overlap of motif M4 and M6 with significant p-value (≤0.01); all 8 sequences belong to actin gene (right panel). (JPG 86 kb) [file 12864_2019_5570_MOESM11_ESM.jpg]

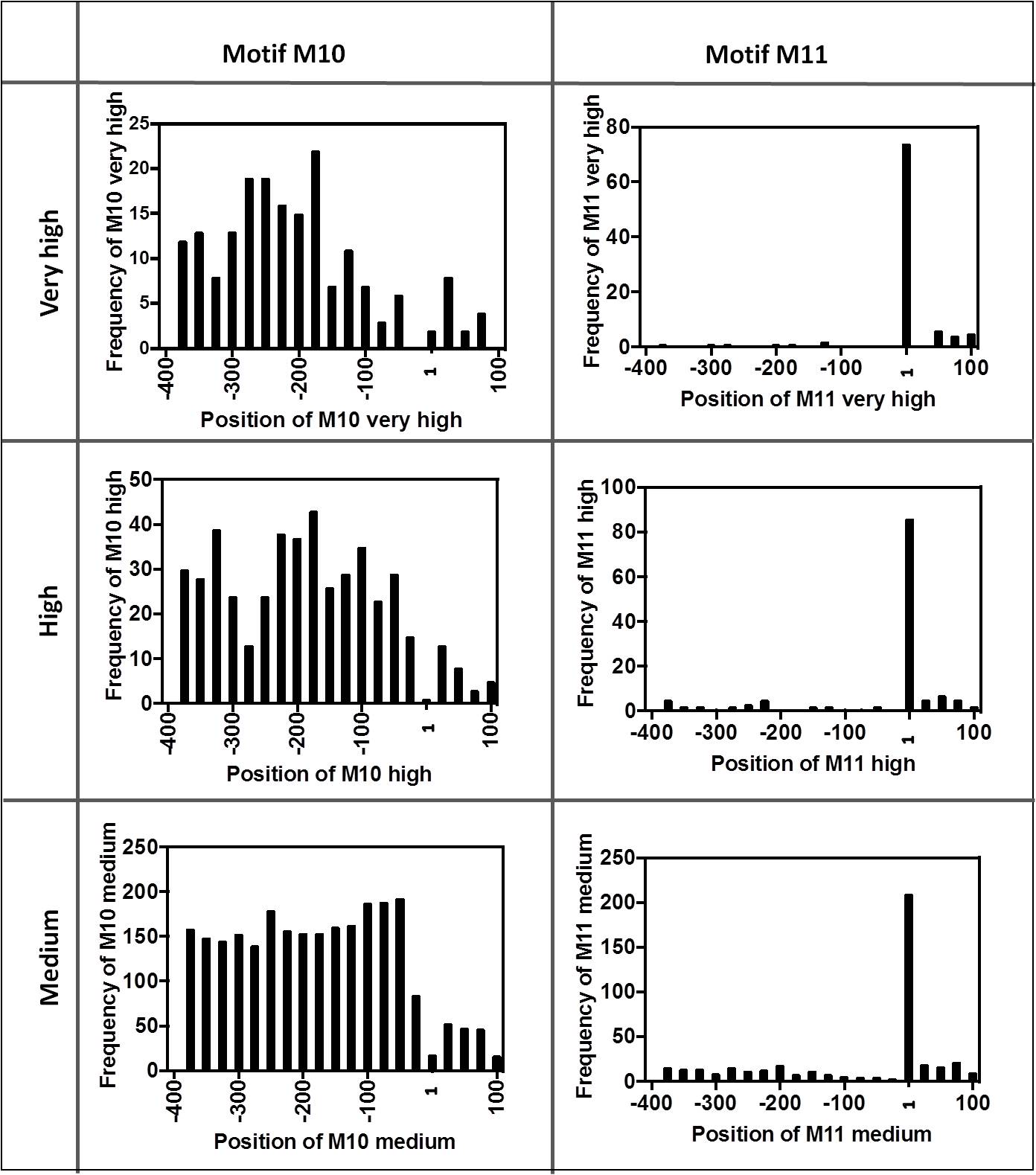

Supplement: Supplementary file 12 — Positions of motifs M10 and M11 with respect to AUG (A = 1). (JPG 208 kb) [file 12864_2019_5570_MOESM12_ESM.jpg]

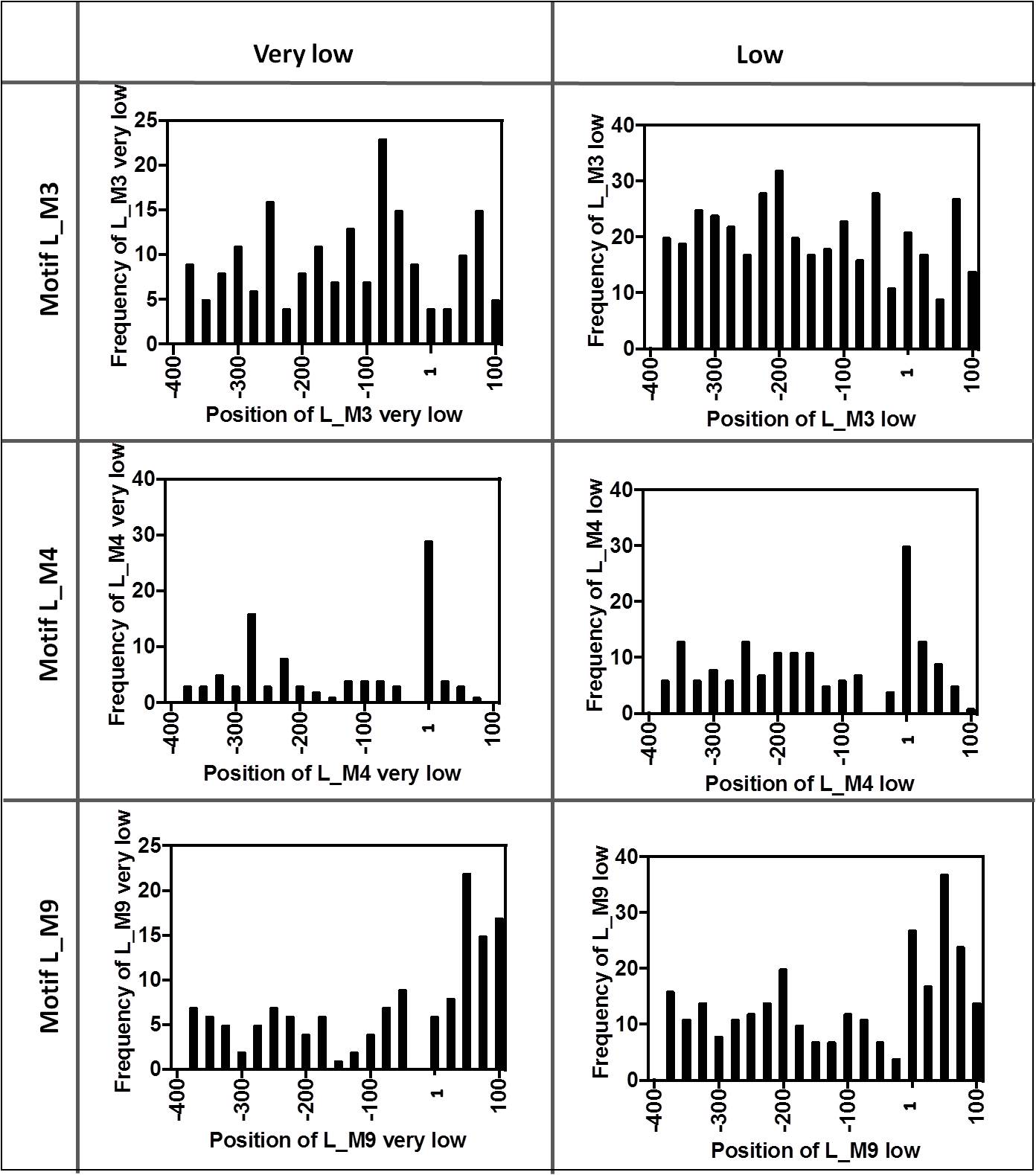

Supplement: Supplementary file 13 — Positions of motifs L_M3, L_M4 and L_M9 with respect to AUG (A = 1). (JPG 212 kb) [file 12864_2019_5570_MOESM13_ESM.jpg]

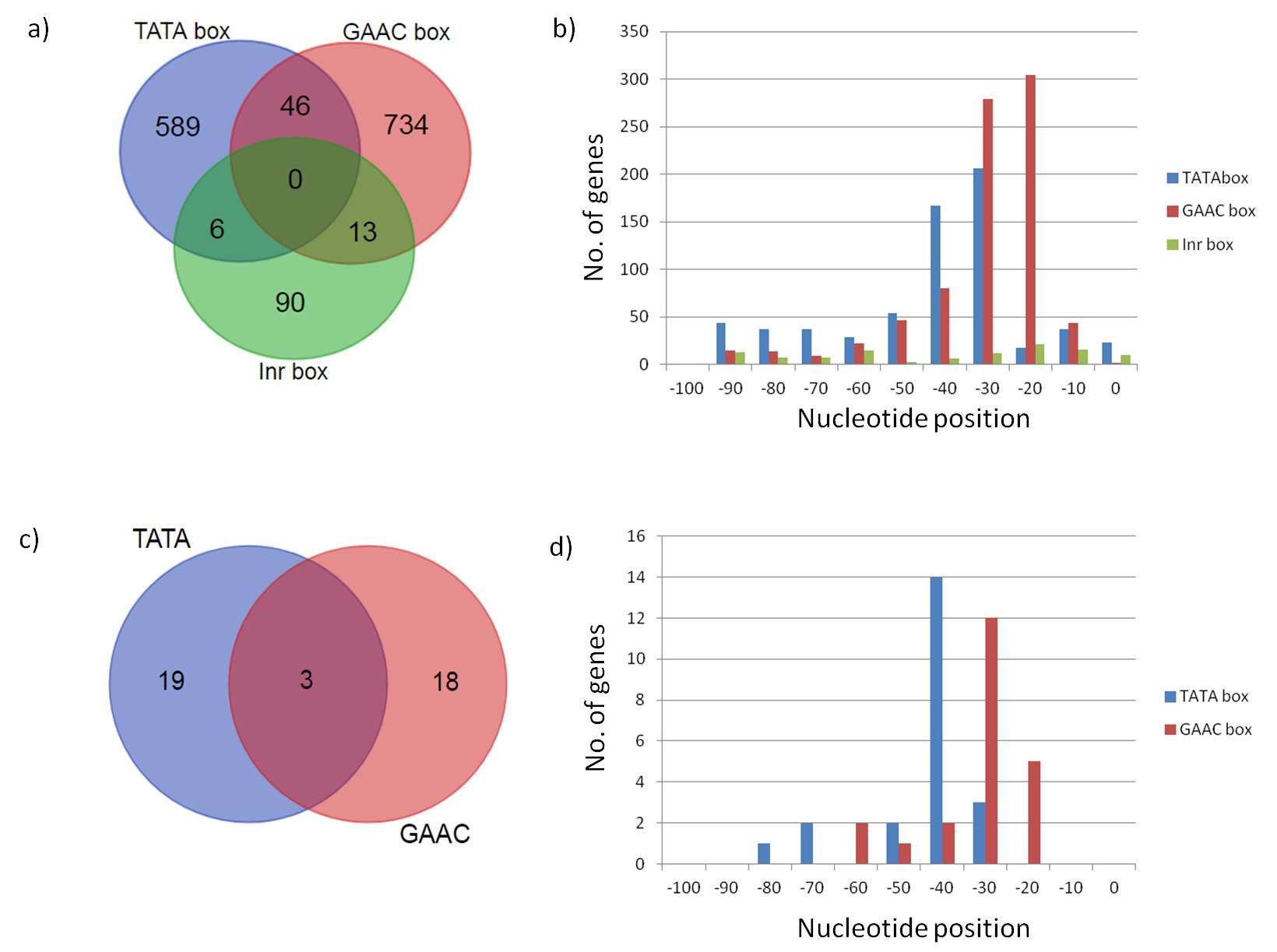

Supplement: Supplementary file 15 — In silico identification of core promoter elements in E. histolytica promoters. (a) Venn diagram displaying the total number for genes having each element and their co-occurrence. (b) Positions of core promoter motifs. No sharp peak was seen for Inr motif owing to its comparatively low number (p value: 0.0001). (c) Core promoter motifs in genes with downstream motifs (M4 and M6). (d) conserved position in these genes (p-value: 0.0001). (JPG 107 kb) [file 12864_2019_5570_MOESM15_ESM.jpg]
