# Supplementary material for: Transcriptomic analysis reveals novel downstream regulatory motifs and highly transcribed virulence factor genes of Entamoeba histolytica
Source: BMC Genomics. 2019 Mar 12;20:206. doi: 10.1186/s12864-019-5570-z (PMC6416950; doi:10.1186/s12864-019-5570-z)
Supplement: Supplementary file 6 — List of genes belonging to very high class. (DOCX 21 kb) [file 12864_2019_5570_MOESM6_ESM.docx]

**Additional file 6:** List of genes belonging to very high class

| **Gene Category** | **Gene name** | **AmoebaDB_ID** | **Log_2_ TPM** |
| --- | --- | --- | --- |
| Ribosomal | 175 genes | ---- | ---- |
| Hypothetical | 44 genes | ---- | ---- |
| Signaling factors | Rab family GTPase (Small GTPase Rab1A) | EHI_108610 | 11.48 |
|  | Rab GDP dissociation inhibitor alpha, putative | EHI_167060 | 11.49 |
|  | Ran family GTPase | EHI_148190 | 11.49 |
|  | Ras family GTPase | EHI_058090 | 11.49 |
|  | Guanine nucleotide-binding protein subunit beta 2-like 1, putative | EHI_050550 | 11.00 |
|  | Guanine nucleotide-binding protein subunit beta 2-like 1, putative | EHI_110400 | 11.00 |
|  | Rho family GTPase | EHI_129750 | 11.49 |
|  | Rho family GTPase | EHI_197840 | 11.49 |
|  | Rho GDP exchange inhibitor, putative | EHI_147570 | 11.50 |
|  | serine-rich protein | EHI_116360 | 11.65 |
|  | Small GTPase Rab7A | EHI_192810 | 11.68 |
|  | Protein tyrosine phosphatase, putative | EHI_110570 | 11.41 |
| Virulence factor | Nonpathogenic pore-forming peptide, putative | EHI_169350 | 11.23 |
|  | Grainin 1 | EHI_167300 | 10.99 |
|  | Grainin 2 | EHI_167310 | 11.00 |
|  | 20 kDa antigen | EHI_057670 | 9.04 |
|  | Amoebapore C | EHI_118270 | 10.75 |
|  | pore-forming peptide ameobapore A precursor 2C putative | EHI_159480 | 11.35 |
|  | pore-forming peptide ameobapore B precursor 2C putative | EHI_194540 | 11.38 |
|  | Cysteine proteinase (EC 3.4.22.35) (Cysteine proteinase, putative) | EHI_168240 | 10.84 |
|  | Cysteine proteinase 1 2C putative | EHI_074180 | 10.85 |
|  | Cysteine proteinase 2 | EHI_033710 | 10.85 |
|  | Galactose-inhibitable lectin 35 kDa subunit | EHI_035690 | 10.98 |
|  | Galactose-specific adhesin light subunit, putative | EHI_049690 | 10.99 |
|  | Gal/GalNAc lectin light subunit | EHI_148790 | 10.96 |
|  | Lysozyme, putative | EHI_096570 | 11.12 |
|  | C2 domain containing protein | EHI_059860 | 10.76 |
|  | High mobility group (HMG) box domain containing protein | EHI_086110 | 11.01 |
| Translation associated | Transcription factor BTF3, putative | EHI_198870 | 11.76 |
|  | Eukaryotic translation elongation factor 1 gamma, putative | EHI_119540 | 10.90 |
|  | Eukaryotic translation initiation factor 5A (eIF-5A) | EHI_151810 | 10.92 |
|  | Eukaryotic translation initiation factor 5A (eIF-5A) | EHI_186480 | 10.92 |
|  | Elongation factor 1 beta, putative (Uncharacterized protein) | EHI_146390 | 10.88 |
|  | Elongation factor 1-alpha | EHI_011210 | 10.88 |
|  | Elongation factor 1-alpha | EHI_102170 | 10.89 |
| Transport factors | Protein transport protein SEC61 gamma subunit, putative | EHI_048170 | 11.41 |
|  | Sec61 alpha subunit, putative | EHI_164500 | 11.65 |
| Calcium ion binding | Calreticulin, putative | EHI_136160 | 10.80 |
|  | Calcium binding protein 2 (Calmodulin, putative) | EHI_010020 | 10.78 |
|  | Calmodulin, putative | EHI_023500 | 10.80 |
|  | EF-hand calcium-binding domain containing protein | EHI_060740 | 10.85 |
| Glycolytic pathway | Acetyl-CoA synthetase, putative | EHI_178960 | 10.54 |
|  | Purine nucleoside phosphorylase, putative | EHI_130930 | 11.41 |
|  | Purine nucleoside phosphorylase, putative | EHI_130960 | 11.41 |
|  | Phosphoglycerate kinase (EC 2.7.2.3) | EHI_188180 | 11.33 |
|  | Galactokinase, putative | EHI_094100 | 10.97 |
|  | Fructose-1,6-bisphosphate aldolase, putative | EHI_098570 | 10.95 |
|  | Enolase, putative | EHI_130700 | 10.90 |
|  | N-acetylmuraminidase, putative | EHI_176820 | 11.14 |
| Cytoskeletal protein binding | Actin (Actin, putative) | EHI_107290 | 10.54 |
|  | Actin (Actin, putative) | EHI_159150 | 10.54 |
|  | Actin (Actin, putative) | EHI_142730 | 10.56 |
|  | Actin (Actin, putative) | EHI_182900 | 10.57 |
|  | Actin (Actin, putative) | EHI_126190 | 10.58 |
|  | Actin (Actin, putative) | EHI_163750 | 10.58 |
|  | Actin (Actin, putative) | EHI_140120 | 10.59 |
|  | Actin 2 protein 2C putative | EHI_161200 | 10.59 |
|  | actin-binding protein 2C cofilin 2F tropomyosin family | EHI_168340 | 10.61 |
|  | Actin-binding protein, cofilin/tropomyosin family | EHI_186840 | 10.61 |
|  | Actobindin, putative | EHI_158570 | 10.62 |
|  | Actobindin, putative | EHI_039020 | 10.62 |
|  | Actophorin, putative | EHI_197480 | 10.63 |
|  | Profilin 2C putative | EHI_176140 | 11.41 |
| Oxidation-reduction | Thioredoxin | EHI_004490 | 11.71 |
|  | Thioredoxin reductase (EC 1.8.1.9) | EHI_155440 | 11.73 |
|  | Thioredoxin, putative | EHI_062790 | 11.73 |
|  | Thioredoxin, putative | EHI_170420 | 11.74 |
|  | Thioredoxin, putative | EHI_026340 | 11.75 |
|  | NADP-dependent alcohol dehydrogenase | EHI_023110 | 11.22 |
|  | Glyceraldehyde-3-phosphate dehydrogenase (EC 1.2.1.12) | EHI_167320 | 10.99 |
|  | Alcohol dehydrogenase 3, putative (Alcohol dehydrogenase, putative) | EHI_198760 | 10.68 |
|  | Alcohol dehydrogenase, putative | EHI_125950 | 10.70 |
|  | Glyceraldehyde-3-phosphate dehydrogenase | EHI_187020 | 9.02 |
|  | 3-oxo-5-alpha-steroid 4-dehydrogenase domain-containing protein (Steroid 5-alpha reductase, putative) | EHI_076870 | 9.07 |
|  | Malate dehydrogenase, putative | EHI_165350 | 11.12 |
|  | Peroxiredoxin | EHI_201250 | 11.26 |
|  | Peroxiredoxin | EHI_001420 | 11.27 |
|  | Peroxiredoxin | EHI_123390 | 11.29 |
|  | Peroxiredoxin | EHI_061980 | 11.29 |
|  | Peroxiredoxin (Peroxiredoxin, putative) | EHI_145840 | 11.31 |
|  | Peroxiredoxin, putative | EHI_122310 | 11.32 |
|  | Superoxide dismutase | EHI_159160 | 11.68 |
|  | Pyruvate phosphate dikinase | EHI_009530 | 11.43 |
|  | Pyruvate, phosphate dikinase, putative | EHI_045080 | 11.45 |
|  | Pyruvate:ferredoxin oxidoreductase | EHI_051060 | 11.48 |
|  | Malic enzyme (Malic enzyme, putative) | EHI_044970 | 11.13 |
| Proteolysis | Ubiquitin (Ubiquitin, putative) | EHI_178340 | 11.88 |
|  | Ubiquitin 2C putative | EHI_156660 | 11.88 |
|  | Ubiquitin-like, putative | EHI_103510 | 11.89 |
|  | Aminoacyl-histidine dipeptidase, putative | EHI_042170 | 10.74 |
| Centromeric DNA binding | Histone H2A | EHI_188960 | 11.03 |
|  | Histone H2B | EHI_182990 | 11.05 |
|  | Histone H2B | EHI_188730 | 11.06 |
|  | Histone H3 2C putative | EHI_135080 | 11.07 |
|  | Histone H3, putative | EHI_096650 | 11.08 |
|  | Histone H4 | EHI_023230 | 11.08 |
| Proteins domain specific binding | 14-3-3 protein 2 | EHI_098280 | 9.02 |
|  | 14-3-3 protein 3 | EHI_006810 | 9.03 |
| Misc. | LIM zinc finger domain containing protein | EHI_194520 | 11.10 |
|  | LIM zinc finger domain containing protein | EHI_069060 | 11.11 |
|  | LIM zinc finger domain containing protein | EHI_096420 | 11.12 |
|  | LIM zinc finger domain containing protein | EHI_030800 | 11.12 |
|  | Polyadenylate-binding protein, putative | EHI_033250 | 11.34 |
|  | PCTP-like protein, putative | EHI_110720 | 11.23 |
|  | Peptidyl-prolyl cis-trans isomerase (PPIase) (EC 5.2.1.8) | EHI_125840 | 11.25 |
|  | Ferredoxin, putative | EHI_198670 | 10.92 |
|  | V-SNARE protein VampD3 (Vesicle-associated membrane protein, putative) | EHI_081370 | 14.03 |
|  | V-type ATPase, C subunit, putative | EHI_059840 | 14.68 |
|  | Enhancer binding protein-2, putative | EHI_182670 | 10.90 |
|  | Endoribonuclease L-PSP, putative | EHI_087570 | 10.89 |
|  | Alpha-NAC protein, putative | EHI_083250 | 10.70 |
