# Supplementary material for: Transcriptomic analysis reveals novel downstream regulatory motifs and highly transcribed virulence factor genes of Entamoeba histolytica
Source: BMC Genomics. 2019 Mar 12;20:206. doi: 10.1186/s12864-019-5570-z (PMC6416950; doi:10.1186/s12864-019-5570-z)
Supplement: Supplementary file 7 — List of genes belonging to very low class. (DOCX 23 kb) [file 12864_2019_5570_MOESM7_ESM.docx]

**Additional file 7:** List of genes belonging to very low class

| **Gene Category** | **Gene name** | **AmoebaDB_ID** | **Log_2_ TPM** |
| --- | --- | --- | --- |
| Hypothetical | 278 genes | ---- | ---- |
| Ribosomal | 40S ribosomal protein S16, putative | EHI_132530 | -7.64 |
|  | rRNA biogenesis protein RRP5, putative | EHI_124060 | -5.32 |
|  | rRNA biogenesis protein RRP5, putative | EHI_160440 | -5.32 |
|  | 40S ribosomal protein S4, putative (Ribosomal protein S4, putative) | EHI_138650 | -7.64 |
|  | 40S ribosomal protein S6 | EHI_011170 | -7.64 |
|  | 60S ribosomal protein L37a, putative | EHI_145340 | -7.64 |
|  | U3 small nucleolar ribonucleoprotein protein IMP4, putative | EHI_072470 | -5.06 |
| Signaling | ATP-binding cassette, putative | EHI_178050 | -7.64 |
|  | ATP-dependent RNA helicase DDX39, putative | EHI_150160 | -7.64 |
|  | ATP/GTP-binding protein, putative | EHI_135320 | -7.64 |
|  | Sodium/calcium exchanger protein, putative | EHI_028330 | -5.32 |
|  | Cyclin, putative | EHI_045020 | -7.64 |
|  | Serine/threonine-protein phosphatase | EHI_174590 | -5.32 |
|  | Serine/threonine-protein phosphatase PP-Z, putative | EHI_076810 | -5.32 |
|  | Signal recognition particle 19 kDa protein, putative | EHI_096300 | -5.32 |
|  | Small GTPase RhoQ, putative | EHI_018960 | -5.32 |
|  | EhRab7H protein (Small GTPase Rab7, putative) | EHI_086890 | -6.64 |
|  | Rho family GTPase | EHI_046630 | -5.32 |
|  | Rab family GTPase (Small GTPase EhRabM2) (Fragment) | EHI_053220 | -5.32 |
|  | Rab family GTPase (Small GTPase EhRabX20) | EHI_041170 | -5.32 |
|  | Rap/Ran GTPase-activating protein, putative | EHI_170950 | -5.32 |
|  | Rap/Ran GTPase-activating protein, putative | EHI_005560 | -5.32 |
|  | GTP-binding protein EhRabX29, putative (Small GTPase EhRabX29) (Fragment) | EHI_184670 | -6.64 |
| Kinases family | Tyrosine kinase, putative | EHI_009580 | -5.06 |
|  | Tyrosine kinase, putative | EHI_180320 | -5.06 |
|  | Tyrosine kinase, putative | EHI_119250 | -5.06 |
|  | Tyrosine kinase, putative | EHI_184810 | -5.06 |
|  | Tyrosine kinase, putative | EHI_110650 | -5.06 |
|  | Tyrosine kinase, putative | EHI_123840 | -5.06 |
|  | Tyrosine kinase, putative (Fragment) | EHI_026890 | -5.06 |
|  | Serine/threonine kinase, putative | EHI_057440 | -5.32 |
|  | Serine/threonine-protein kinase PLK (EC 2.7.11.21) (Polo-like kinase) | EHI_142120 | -5.32 |
|  | Protein kinase domain containing protein | EHI_034210 | -5.64 |
|  | Protein kinase domain containing protein | EHI_000270 | -5.64 |
|  | Protein kinase domain containing protein | EHI_195370 | -5.64 |
|  | Protein kinase domain containing protein | EHI_062090 | -5.64 |
|  | Protein kinase domain containing protein | EHI_103240 | -5.64 |
|  | Protein kinase domain containing protein | EHI_120930 | -5.64 |
|  | Protein kinase domain containing protein | EHI_059040 | -5.64 |
|  | Protein kinase domain containing protein | EHI_144590 | -5.64 |
|  | Protein kinase domain containing protein | EHI_064610 | -5.64 |
|  | Protein kinase, putative | EHI_092300 | -5.64 |
|  | Protein kinase, putative | EHI_185130 | -5.64 |
|  | Protein kinase, putative | EHI_184540 | -5.64 |
|  | Protein kinase, putative | EHI_067070 | -5.64 |
|  | Protein kinase, putative | EHI_066510 | -5.64 |
|  | Protein kinase, putative | EHI_051910 | -5.64 |
|  | Protein kinase, putative | EHI_193670 | -5.64 |
|  | Protein kinase, putative | EHI_157150 | -5.64 |
|  | Protein kinase, putative | EHI_175920 | -5.64 |
|  | Protein kinase, putative | EHI_196510 | -5.64 |
|  | Protein kinase, putative (Serine-threonine protein kinase PK2) | EHI_064920 | -5.64 |
|  | Thymidylate kinase, putative | EHI_132870 | -5.06 |
| mRNA catabolic process | Regulator of nonsense transcripts, putative | EHI_110840 | -5.32 |
|  | Regulator of nonsense transcripts, putative | EHI_193520 | -5.32 |
|  | Regulator of nonsense transcripts, putative | EHI_070810 | -5.32 |
| Virulence factor | Cysteine protease, putative | EHI_097900 | -7.64 |
|  | Cysteine protease, putative | EHI_121160 | -7.64 |
|  | Cysteine protease, putative (Fragment) | EHI_180650 | -7.64 |
|  | Cysteine surface protein, putative | EHI_160750 | -7.64 |
|  | Cysteine surface protein, putative | EHI_030860 | -7.64 |
|  | Cysteine surface protein, putative | EHI_091840 | -7.64 |
|  | AIG1 family protein | EHI_067730 | -7.64 |
|  | AIG1 family protein | EHI_136940 | -7.64 |
|  | AIG1 family protein | EHI_115150 | -7.64 |
|  | AIG1 family protein, putative | EHI_136950 | -7.64 |
|  | AIG1 family protein, putative | EHI_119040 | -7.64 |
|  | Surface antigen ariel1, putative | EHI_081010 | -5.06 |
|  | Surface antigen ariel1, putative | EHI_005260 | -5.06 |
|  | Surface antigen ariel1, putative (Fragment) | EHI_041270 | -5.06 |
|  | Myb-like DNA-binding domain containing protein | EHI_009930 | -6.06 |
| Translation associated | Eukaryotic translation initiation factor 2 alpha subunit, putative | EHI_078520 | -6.64 |
|  | Elongation factor 1-alpha | EHI_195770 | -6.64 |
| DNA-directed DNA polymerase | DNA polymerase (EC 2.7.7.7) | EHI_164190 | -6.64 |
|  | DNA polymerase, putative | EHI_018010 | -6.64 |
|  | DNA polymerase, putative | EHI_132860 | -6.64 |
|  | DNA polymerase, putative | EHI_196700 | -6.64 |
| Integral membrane component | Tubulin gamma chain, putative | EHI_139330 | -5.06 |
|  | F-actin capping protein beta subunit, putative | EHI_074600 | -6.64 |
|  | Cell surface protease gp63, putative | EHI_042870 | -7.64 |
|  | Glycosyltransferase, putative | EHI_151870 | -6.64 |
|  | P-glycoprotein-2, putative | EHI_001290 | -5.64 |
|  | P-glycoprotein-2, putative | EHI_024540 | -5.64 |
| Transport factors | Sec1 family domain containing protein | EHI_108480 | -5.32 |
|  | EhSly1 (Sec1 family protein) | EHI_018150 | -6.64 |
|  | Transporter, major facilitator family | EHI_135920 | -5.06 |
|  | Amino acid transporter, putative | EHI_190460 | -7.64 |
|  | EhSyntaxin 1B (Syntaxin, putative) (Fragment) | EHI_052830 | -6.64 |
|  | Sec7 domain protein | EHI_053470 | -5.32 |
| Cell metabolism | 78 kD aglucose-regulated protein homolog, putative | EHI_104000 | -7.64 |
|  | Acyl-CoA synthetase, putative | EHI_153060 | -7.64 |
|  | Alcohol dehydrogenase, putative | EHI_005050 | -7.64 |
|  | Ribulose-phosphate 3-epimerase (EC 5.1.3.1) | EHI_111950 | -5.32 |
|  | Protein-S-isoprenylcysteine O-methyltransferase (EC 2.1.1.100) | EHI_094580 | -5.32 |
|  | Deoxyuridine 5’-triphosphate nucleotidohydrolase domain containing protein | EHI_193660 | -6.64 |
|  | Deoxyuridine 5’-triphosphate nucleotidohydrolase domain containing protein | EHI_116940 | -6.64 |
|  | Dextranase, putative | EHI_182460 | -6.64 |
|  | Transketolase, putative | EHI_036390 | -5.06 |
|  | Palmitoyltransferase (EC 2.3.1.225) | EHI_074520 | -5.64 |
|  | Dentin sialophospho protein, putative | EHI_037010 | -6.64 |
|  | Dentin sialophospho protein, putative | EHI_188600 | -6.64 |
|  | Malic enzyme, putative | EHI_050330 | -6.06 |
|  | Mannose-6-phosphate isomerase, putative | EHI_034600 | -6.06 |
|  | Metallo-beta-lactamase superfamily protein | EHI_115720 | -6.06 |
|  | Threonylcarbamoyl-AMP synthase (TC-AMP synthase) (EC 2.7.7.87) (L-threonylcarbamoyl adenylate synthase) | EHI_153810 | -5.06 |
|  | Fe-hydrogenase, putative (Putative long iron-dependent hydrogenase 2) | EHI_005060 | -6.64 |
| Nucleotide binding | Chromodomain-helicase-DNA-binding protein, putative | EHI_017350 | -7.64 |
|  | Nucleotide binding protein 2, putative | EHI_011990 | -5.64 |
|  | Chaperone clpB, putative | EHI_090840 | -7.64 |
|  | DNA mismatch repair protein mutL, putative | EHI_126120 | -6.64 |
|  | Splicing factor3B subunit 1, putative (Fragment) | EHI_085470 | -5.06 |
|  | Endonuclease/exonuclease/phosphatase family protein (Phospholipase C, putative) | EHI_123700 | -6.64 |
|  | tRNA intron endonuclease, putative | EHI_092410 | -5.06 |
| Heat shock proteins | Heat shock protein 101, putative (Fragment) | EHI_076480 | -6.64 |
|  | Heat shock protein 70 family (Fragment) | EHI_112590 | -6.64 |
|  | Heat shock protein 70, putative | EHI_113410 | -6.64 |
|  | Heat shock protein, putative | EHI_042860 | -6.64 |
|  | Heat shock protein, putative | EHI_034710 | -6.64 |
|  | Heat shock protein, putative | EHI_022620 | -6.64 |
|  | Heat shock protein70, hsp70A2, putative | EHI_192440 | -6.64 |
|  | Heat shock transcription factor, putative | EHI_145450 | -6.64 |
| Bsp family | Leucine rich repeat / protein phosphatase 2C domain containing protein | EHI_087420 | -6.64 |
|  | Leucine rich repeat protein, BspA family | EHI_018840 | -6.64 |
|  | Leucine rich repeat protein, BspA family | EHI_003380 | -6.64 |
|  | Leucine rich repeat protein, BspA family | EHI_066620 | -6.64 |
|  | Leucine rich repeat protein, BspA family | EHI_113190 | -6.64 |
|  | Leucine rich repeat protein, BspA family | EHI_077280 | -6.64 |
|  | Leucine rich repeat protein, BspA family | EHI_049160 | -6.64 |
|  | Leucine rich repeat protein, BspA family | EHI_189090 | -6.64 |
|  | Leucine rich repeat protein, BspA family | EHI_184260 | -6.06 |
|  | Leucine rich repeat protein, BspA family | EHI_041470 | -6.06 |
|  | Leucine rich repeat protein, BspA family | EHI_120570 | -6.06 |
|  | Leucine rich repeat protein, BspA family | EHI_103140 | -6.06 |
|  | Leucine rich repeat protein, BspA family | EHI_100700 | -6.06 |
|  | Leucine rich repeat protein, BspA family | EHI_112290 | -6.06 |
|  | Leucine rich repeat protein, BspA family | EHI_128460 | -6.06 |
|  | Leucine rich repeat protein, BspA family | EHI_166160 | -6.06 |
|  | Leucine rich repeat protein, BspA family | EHI_176480 | -6.06 |
|  | Leucine rich repeat protein, BspA family | EHI_070330 | -6.06 |
|  | Leucine rich repeat protein, BspA family | EHI_124070 | -3.00 |
|  | Leucine rich repeat protein, BspA family | EHI_160700 | -5.32 |
|  | Leucine rich repeat protein, BspA family | EHI_137910 | -5.32 |
|  | Leucine rich repeat protein, BspA family | EHI_123820 | -6.06 |
|  | Leucine rich repeat protein, BspA family | EHI_070440 | -6.06 |
|  | Leucine rich repeat protein, BspA family (Fragment) | EHI_105370 | -6.06 |
| Proteolysis | Ubiquitin carboxyl-terminal hydrolase, putative | EHI_154590 | -5.06 |
|  | Proteasome subunit alpha type (EC 3.4.25.1) | EHI_062360 | -5.64 |
|  | Peptidase, C54 family | EHI_057950 | -5.64 |
| Zn finger domain | LIM zinc finger domain containing protein | EHI_047620 | -6.06 |
|  | Zinc finger protein, putative | EHI_148010 | -3.00 |
| Misc. | Ankyrin repeat protein, putative | EHI_128800 | -7.64 |
|  | CXXC-rich protein | EHI_082260 | -7.64 |
|  | Rodhanase-like domain containing protein | EHI_122410 | -5.32 |
|  | Serine-rich 25 kDa antigen protein, putative | EHI_072000 | -5.32 |
|  | Serine-threonine-isoleucine rich protein, putative | EHI_012330 | -5.32 |
|  | Serine-threonine-isoleucine rich protein, putative | EHI_073630 | -5.32 |
|  | Repeat organellar protein, putative | EHI_091710 | -5.32 |
|  | Hybrid-cluster protein (Hydroxylamine reductase, putative) | EHI_060210 | -6.64 |
|  | Interaptin, putative | EHI_173620 | -6.64 |
|  | Iron-sulfur flavoprotein, putative | EHI_135180 | -6.64 |
|  | G-box-binding factor, putative (Fragment) | EHI_185110 | -6.64 |
|  | F-box domain containing protein | EHI_049810 | -6.64 |
|  | Dr1-associated corepressor, putative | EHI_160460 | -6.64 |
|  | PQ loop repeat protein | EHI_187250 | -5.64 |
|  | Thioredoxin, putative | EHI_104650 | -5.06 |
|  | Trichohyalin, putative | EHI_077870 | -5.06 |
