# Supplementary material for: Transcriptomic analysis reveals novel downstream regulatory motifs and highly transcribed virulence factor genes of Entamoeba histolytica
Source: BMC Genomics. 2019 Mar 12;20:206. doi: 10.1186/s12864-019-5570-z (PMC6416950; doi:10.1186/s12864-019-5570-z)
Supplement: Supplementary file 16 — Status of cysteine protease expression in comparison to Tillack et al., 2007 and Matthiesen et al., 2013 [35,36]. (DOCX 20 kb) [file 12864_2019_5570_MOESM16_ESM.docx]

**Additional file 16:** Status of cysteine protease expression in comparison to Tillack *et al.*, 2007 and Matthiesen *et al.*, 2013 (36,37).

| **S.No** | **Gene_ID** | **Annotation** | **Log_2_ Normal** | **Class** | **Motifs** | **Location** | **Ref(36,37)** | |
| --- | --- | --- | --- | --- | --- | --- | --- | --- |
|  |  |  |  |  |  |  | **Name** | **Expression** |
| 1 | EHI_033710 | Cysteine proteinase 2 | 13.34 | VH | --- | --- | CPA2 | High |
| 2 | EHI_074180 | Cysteine proteinase 1 | 12.66 | VH | M10 | -56 to -45 | CPA1 | High |
| 3 | EHI_168240 | Cysteine proteinase | 10.85 | VH | M1 | -223 to -212 | CPA5 | High |
| 4 | EHI_010850 | Cysteine proteinase, putative | 8.48 | H | --- | --- | --- | --- |
| 5 | EHI_039610 | Cysteine protease 8 | 6.79 | H | --- | --- | CPA7 | --- |
| 6 | EHI_197490 | Cysteine protease 19 | 6.79 | H | M1 | -86 to -75, -80 to -69 | CPA11 | Intermediate |
|  |  |  |  |  | M10 | -370 to -359, -367 to -356 |  |  |
| 7 | EHI_050570 | Cysteine proteinase | 5.77 | M | M1 | -219 to -208 | CPA4 | Low |
| 8 | EHI_151440 | Cysteine proteinase | 5.24 | M | M11 | 63 to 73 | CPA6 | Intermediate |
| 9 | EHI_138460 | Papain family cysteine protease domain containing protein | 5.39 | M | M10 | -110 to -99, -93 to -82 | CPC3 | Low |
| 10 | EHI_030720 | Cysteine protease 13 | 5.09 | M | --- | --- | CPB4 | Low |
| 11 | EHI_045290 | Calpain family cysteine protease | 4.77 | M | M5 | -24 to -14 | --- | Low |
| 12 | EHI_181230 | Cysteine protease | 3.37 | M | M10 | -307 to -296, -262 to -254 | CPB9 | Low |
| 13 | EHI_062480 | Cysteine protease 17 | 3.35 | M | M1 | -375 to -361, -348 to -337, -312 to -301, | CPA10 | Intermediate |
|  |  |  |  |  | M5 | -41 to -31 |  |  |
| 14 | EHI_064430 | OTU-like cysteine protease | 3.02 | M | M10 | -249 to -238 | --- | Low |
| 15 | EHI_006920 | Papain family cysteine protease domain containing protein | 2.69 | M | M5 | -40 to -30 | CPC1 | Low |
| 16 | EHI_151400 | Cysteine protease 9 | 2.68 | M | M5 | -29 to -19 | CPA8 | Low |
| 17 | EHI_200690 | Cysteine protease 14 | 1.94 | M | M10 | -341 to -330 | CPB5 | Low |
| 18 | EHI_180170 | Cysteine protease | 2.09 | M | M10 | -349 to -338 | --- | Low |
| 19 | EHI_096740 | Cysteine protease 10 | 1.44 | M | M10 | -252 to -241, 15 to 26 | CPA9 | Low |
| 20 | EHI_108240 | Cysteine protease, putative | -0.84 | L | --- | --- | --- | --- |
| 21 | EHI_140220 | Cysteine protease 12 | -0.16 | L | M1 | -29 to -18,-159 to -148,-30 to -19,-31 to -20, -227 to -217  -79 to -68 | CPB3 | Low |
|  |  |  |  |  | M5 |  |  |  |
|  |  |  |  |  | L_M3 |  |  |  |
| 22 | EHI_091450 | Cysteine protease 16 | 0.33 | L | M1 | -380 to -369 | CPB7 | Low |
| 23 | EHI_117650 | Cysteine protease 7 | -1.25 | L | M5 | -19 to -09 | CPB1 | Low |
| 24 | EHI_159610 | Cysteine protease | -2.36 | L | M1 | -197 to -186, -386 to -375,-196 to -185,191 to -180,-193 to -182,-384 to -373,-383 to -372, -195 to -184 | CPA3 | Low |
| 25 | EHI_126170 | Cysteine protease 15 | -2.25 | L | L_M4 | -83 to -92 | --- | Low |
| 26 | EHI_121160 | Cysteine protease, putative | -3.74 | VL | L_M3 | -397 to -386 | --- | --- |
| 27 | EHI_180650 | Cysteine protease | -4.18 | VL | --- | --- | CPB10 | Low |
| 28 | EHI_097900 | Cysteine protease 18 | -7.64 | VL | --- | --- | CPB8 | Low |
