# Supplementary material for: Transcriptomic analysis reveals novel downstream regulatory motifs and highly transcribed virulence factor genes of Entamoeba histolytica
Source: BMC Genomics. 2019 Mar 12;20:206. doi: 10.1186/s12864-019-5570-z (PMC6416950; doi:10.1186/s12864-019-5570-z)
Supplement: Supplementary file 17 — Up regulated genes in serum starved cells. (Of the 185 up regulated genes 93 were uncharacterized). (DOCX 23 kb) [file 12864_2019_5570_MOESM17_ESM.docx]

**Additional file 17: Up regulated genes in serum starved cells. (**Of the 185 up regulated genes 93 were uncharacterized**)**

| **S.No** | **Gene** | **Log_2_ (FC)** | **Class** | **Annotation** | **Log_2_ TPM** |
| --- | --- | --- | --- | --- | --- |
| 1 | EHI_189960 | 5.83 | M | ADP-ribosylation factor 1, putative (ADP-ribosylation factor, putative) (Small GTPase ArfA2) | 5.39 |
| 2 | EHI_154270 | 4.27 | L | Cell division control protein 42, putative | -0.92 |
| 3 | EHI_073480 | 3.46 | M | ADP-ribosylation factor, putative (Small GTPase ArfA4) | 4.15 |
| 4 | EHI_009930 | 3.36 | VL | Myb-like DNA-binding domain containing protein | -4.32 |
| 5 | EHI_118110 | 3.28 | L | Acid sphingomyelinase-like phosphodiesterase | -2.12 |
| 6 | EHI_156390 | 3.22 | L | Casein kinase 1, putative | -1.79 |
| 7 | EHI_062750 | 3.15 | L | Leucine rich repeat protein, BspA family | -0.90 |
| 8 | EHI_054720 | 3.09 | L | CDP-alcohol phosphatidyltransferase family protein | 0.08 |
| 9 | EHI_041465 | 2.92 | VL | NA | -3.00 |
| 10 | EHI_066970 | 2.83 | L | Mucin-like protein 1, putative | -0.84 |
| 11 | EHI_194600 | 2.59 | H | Molybdenum cofactor sulfurase putative | 6.29 |
| 12 | EHI_006885 | 2.55 | M | NA | 5.86 |
| 13 | EHI_008130 | 2.51 | M | Myb family DNA-binding protein, SHAQKYF family | 3.59 |
| 14 | EHI_112490 | 2.51 | L | Serine-rich 25 kDa antigen protein, putative | 0.06 |
| 15 | EHI_151800 | 2.47 | H | Ganglioside gm2 activator protein, putative | 6.05 |
| 16 | EHI_022950 | 2.44 | M | DnaJ family protein | 5.76 |
| 17 | EHI_126170 | 2.37 | L | Cysteine protease 15 (Cysteine protease, putative) | -2.25 |
| 18 | EHI_130710 | 2.27 | L | Myb-like DNA-binding domain containing protein | -1.20 |
| 19 | EHI_153710 | 2.23 | M | Methylene-fatty-acyl-phospholipid synthase, putative | 3.24 |
| 20 | EHI_174250 | 1.99 | M | S-adenosylmethionine synthase (EC 2.5.1.6) | 5.70 |
| 21 | EHI_177460 | 1.99 | M | Eukaryotic translation initiation factor 5A (eIF-5A) | 4.03 |
| 22 | EHI_058330 | 1.99 | H | Galactose-inhibitable lectin, putative | 8.70 |
| 23 | EHI_008350 | 1.93 | L | Rab family GTPase (Small GTPase EhRabX6) (Fragment) | 0.71 |
| 24 | EHI_155580 | 1.91 | M | Myb family DNA-binding protein, SHAQKYF family | 2.78 |
| 25 | EHI_121870 | 1.89 | M | ADP ribosylation factor family GTPase, putative (Small GTPase ArfA3) | 2.86 |
| 26 | EHI_020610 | 1.87 | M | TATA-box-binding protein 1 (EhTBP) | 5.46 |
| 27 | EHI_197070 | 1.85 | M | Steroid 5-alpha reductase, putative | 4.73 |
| 28 | EHI_009590 | 1.83 | L | Serine/threonine protein kinase, putative | 0.87 |
| 29 | EHI_126260 | 1.80 | M | WD domain containing protein | 3.91 |
| 30 | EHI_040600 | 1.78 | M | Acid sphingomyelinase-like phosphodiesterase | 3.50 |
| 31 | EHI_105080 | 1.78 | M | Zinc finger protein, putative | 2.64 |
| 32 | EHI_020250 | 1.75 | M | Lecithin:cholesterol acyltransferase domain-containing protein | 3.34 |
| 33 | EHI_079300 | 1.73 | M | Long-chain-fatty-acid CoA ligase, putative | 2.70 |
| 34 | EHI_013170 | 1.72 | M | Protein kinase domain containing protein | 5.46 |
| 35 | EHI_153000 | 1.71 | L | Pyridine nucleotide-disulfide oxidoreductase family protein | 0.03 |
| 36 | EHI_092190 | 1.71 | M | 3-ketoacyl-CoA synthase (EC 2.3.1.-) | 2.59 |
| 37 | EHI_040260 | 1.68 | L | HEAT repeat domain containing protein | 0.77 |
| 38 | EHI_117970 | 1.65 | L | DNA helicase (EC 3.6.4.12) | 0.79 |
| 39 | EHI_182610 | 1.63 | M | Myb-like DNA-binding domain containing protein | 4.31 |
| 40 | EHI_134640 | 1.60 | H | Acetyltransferase, putative | 7.88 |
| 41 | EHI_100480 | 1.58 |  | MIR domain protein | 6.30 |
| 42 | EHI_152430 | 1.56 | M | Cell division protein kinase, putative | 1.33 |
| 43 | EHI_148580 | 1.55 | M | Choline/ethanolamine kinase, putative | 3.84 |
| 44 | EHI_008360 | 1.55 | M | Ankyrin repeat protein, putative | 1.65 |
| 45 | EHI_137090 | 1.53 | M | TFIID subunit, putative | 4.05 |
| 46 | EHI_055680 | 1.53 | M | Heat shock protein, Hsp20 family, putative | 3.03 |
| 47 | EHI_126270 | 1.50 | M | Ras GTPase-activating protein, putative | 2.80 |
| 48 | EHI_182520 | 1.49 | M | DNAJ homolog subfamily A member 1, putative | 5.01 |
| 49 | EHI_054240 | 1.49 | L | DNA repair helicase, putative | -0.02 |
| 50 | EHI_162570 | 1.45 | M | Ornithine cyclodeaminase, putative | 2.98 |
| 51 | EHI_058470 | 1.44 | M | NA | 4.15 |
| 52 | EHI_118640 | 1.43 | M | Negative cofactor 2-beta | 5.23 |
| 53 | EHI_042260 | 1.43 | M | NADP-dependent alcohol dehydrogenase, putative | 3.86 |
| 54 | EHI_008190 | 1.41 | M | RNA recognition motif domain containing protein | 1.62 |
| 55 | EHI_009370 | 1.41 | M | 3-ketoacyl-CoA synthase (EC 2.3.1.-) | 3.88 |
| 56 | EHI_127170 | 1.40 | M | Elongation factor-2 kinase, putative | 2.61 |
| 57 | EHI_158610 | 1.36 | M | Activator 1 140 kDa subunit, putative | 1.83 |
| 58 | EHI_044190 | 1.36 | M | Protein kinase with WD repeats | 1.12 |
| 59 | EHI_151810 | 1.34 | VH | Eukaryotic translation initiation factor 5A (eIF-5A) | 9.84 |
| 60 | EHI_008770 | 1.33 | M | Zinc finger protein, putative | 4.43 |
| 61 | EHI_038330 | 1.31 | M | Zinc finger domain containing protein | 4.98 |
| 62 | EHI_197060 | 1.30 | M | Ser/thr protein phosphatase family protein | 5.11 |
| 63 | EHI_126990 | 1.30 | M | Delta2-COP (Uncharacterized protein) (Fragment) | 3.47 |
| 64 | EHI_025710 | 1.27 | M | Iron-sulfur flavoprotein, putative | 4.11 |
| 65 | EHI_126160 | 1.26 | M | DNA excision repair protein, putative | 2.96 |
| 66 | EHI_143110 | 1.21 | H | Acetyltransferase, putative | 8.65 |
| 67 | EHI_142250 | 1.19 | H | Methionine gamma-lyase | 6.74 |
| 68 | EHI_199590 | 1.17 | H | 70 kDa heat shock protein, putative | 7.40 |
| 69 | EHI_166810 | 1.17 | H | Elongation factor 2 | 6.52 |
| 70 | EHI_020210 | 1.13 | M | Ras guanine nucleotide exchange factor, putative | 3.09 |
| 71 | EHI_009800 | 1.10 | M | Phosphatidylserine synthase, putative | 5.19 |
| 72 | EHI_166440 | 1.10 | M | Zinc finger domain containing protein | 3.92 |
| 73 | EHI_040310 | 1.09 | H | Rab family GTPase (Small GTPase EhRabX31) (Fragment) | 8.79 |
| 74 | EHI_141360 | 1.09 | M | Acetyltransferase, GNAT family | 4.74 |
| 75 | EHI_014000 | 1.07 | M | Retinoblatoma-binding protein 6, putative | 3.18 |
| 76 | EHI_110620 | 1.07 | H | Zinc finger domain containing protein | 6.36 |
| 77 | EHI_194390 | 1.07 | M | Rho family GTPase | 5.50 |
| 78 | EHI_118440 | 1.06 | M | Beta-amylase, putative | 5.14 |
| 79 | EHI_111080 | 1.06 | M | Hsp70 family protein | 2.43 |
| 80 | EHI_080720 | 1.06 | M | Aminoalcoholphosphotransferase, putative | 4.66 |
| 81 | EHI_133950 | 1.04 | M | Heat shock protein 70, putative | 3.98 |
| 82 | EHI_110500 | 1.04 | M | Beta-N-acetylhexosaminidase, putative | 4.96 |
| 83 | EHI_130670 | 1.03 | H | Rab family GTPase (Small GTPase EhRabX35) | 6.25 |
| 84 | EHI_039830 | 1.03 | M | UDP-N-acetylglucosamine pyrophosphorylase | 4.21 |
| 85 | EHI_131490 | 1.02 | M | Leucine rich repeat protein, BspA family | 3.84 |
| 86 | EHI_045610 | 1.01 | M | Diacylglycerol kinase (DAG kinase) | 3.16 |
| 87 | EHI_197530 | 1.01 | M | Sodium/hydrogen exchanger | 2.82 |
| 88 | EHI_023470 | 0.97 | M | Oxysterol binding protein, putative | 5.46 |
| 89 | EHI_160710 | 0.96 | M | Ribonuclease, putative | 5.75 |
| 90 | EHI_045310 | 0.94 | H | PH domain containing protein | 6.17 |
