# Supplementary material for: Transcriptomic analysis reveals novel downstream regulatory motifs and highly transcribed virulence factor genes of Entamoeba histolytica
Source: BMC Genomics. 2019 Mar 12;20:206. doi: 10.1186/s12864-019-5570-z (PMC6416950; doi:10.1186/s12864-019-5570-z)
Supplement: Supplementary file 18 — Down regulated genes in serum starved cells (Of the 157 down regulated genes 48 were uncharacterized). (DOCX 25 kb) [file 12864_2019_5570_MOESM18_ESM.docx]

**Additional file 18: Down regulated genes in serum starved cells (**Of the 157 down regulated genes 48 were uncharacterized**)**

| **S.No** | **Gene** | **Log_2_ (FC)** | **Class** | **Annotation** | **Log_2_ TPM** |
| --- | --- | --- | --- | --- | --- |
| 1 | EHI_161200 | -3.38 | VH | Actin 2 protein, putative (Fragment) | 13.05 |
| 2 | EHI_068110 | -3.31 | L | Ras guanine nucleotide exchange factor, putative | -0.75 |
| 3 | EHI_095820 | -3.23 | M | ATP-binding cassette, putative | 5.37 |
| 4 | EHI_045080 | -3.22 | VH | Pyruvate, phosphate dikinase, putative | 9.30 |
| 5 | EHI_101730 | -2.95 | H | Surface antigen ariel1, putative | 7.10 |
| 6 | EHI_131360 | -2.89 | VH | Surface antigen ariel1, putative | 10.02 |
| 7 | EHI_139080 | -2.89 | M | Longevity-assurance family protein | 1.43 |
| 8 | EHI_039020 | -2.85 | VH | Actobindin, putative | 11.88 |
| 9 | EHI_112870 | -2.75 | M | 3-ketoacyl-CoA synthase | 5.96 |
| 10 | EHI_014030 | -2.63 | H | NAD(P) transhydrogenase subunit alpha, putative (Pyridine nucleotidetranshydrogenase) | 6.70 |
| 11 | EHI_158570 | -2.63 | VH | Actobindin, putative | 12.37 |
| 12 | EHI_093870 | -2.62 | M | Phosphoglycerate mutase domain family protein | 3.50 |
| 13 | EHI_074090 | -2.50 | VH | 40S ribosomal protein S14, putative (Ribosomal protein S14) | 11.49 |
| 14 | EHI_186810 | -2.49 | M | Major facilitator superfamily protein | 1.93 |
| 15 | EHI_105060 | -2.48 | H | Geranylgeranyl pyrophosphate synthase, putative | 7.97 |
| 16 | EHI_165270 | -2.47 | H | L-myo-inositol-1-phosphate synthase, putative | 6.67 |
| 17 | EHI_070720 | -2.45 | H | L-myo-inositol-1-phosphate synthase | 7.89 |
| 18 | EHI_150490 | -2.42 | H | Aldehyde-alcohol dehydrogenase | 8.74 |
| 19 | EHI_160940 | -2.38 | H | Aldehyde-alcohol dehydrogenase | 6.92 |
| 20 | EHI_080200 | -2.30 | VH | Surface antigen ariel1, putative | 9.14 |
| 21 | EHI_157120 | -2.30 | H | Kinase, PfkB family | 6.17 |
| 22 | EHI_156240 | -2.24 | H | Competence protein ComEC, putative | 7.80 |
| 23 | EHI_096710 | -2.18 | VH | Type A flavoprotein, putative | 9.44 |
| 24 | EHI_012360 | -2.18 | VH | 40S ribosomal protein S14, putative (Ribosomal protein S14) | 11.49 |
| 25 | EHI_186470 | -2.18 | VH | Surface antigen ariel1, putative | 9.14 |
| 26 | EHI_084690 | -2.13 | M | Lecithin:cholesterol acyltransferase domain-containing protein | 3.75 |
| 27 | EHI_163240 | -2.03 | M | Phosphatidate cytidylyltransferase, putative | 5.93 |
| 28 | EHI_098180 | -1.93 | H | Surface antigen ariel1, putative | 8.10 |
| 29 | EHI_142720 | -1.93 | M | Bifunctional short chain isoprenyl diphosphate synthase | 5.24 |
| 30 | EHI_172850 | -1.93 | M | Surface antigen ariel1, putative | 5.00 |
| 31 | EHI_151440 | -1.93 | M | Cysteine proteinase, putative | 5.24 |
| 32 | EHI_073380 | -1.92 | M | Carbonic anhydrase (Carbonate dehydratase) | 3.97 |
| 33 | EHI_143580 | -1.83 | M | UMP-CMP kinase (UMP-CMP kinase, putative) | 4.02 |
| 34 | EHI_152650 | -1.82 | VH | Type A flavoprotein, putative | 9.44 |
| 35 | EHI_074180 | -1.79 | VH | Cysteine proteinase 1, putative | 12.66 |
| 36 | EHI_027800 | -1.74 | H | Galactose binding lectin 35 kDa subunit, putative | 7.88 |
| 37 | EHI_141000 | -1.71 | M | Transporter, major facilitator family | 3.86 |
| 38 | EHI_024240 | -1.69 | M | Aldehyde-alcohol dehydrogenase 2, putative | 3.74 |
| 39 | EHI_026360 | -1.68 | M | Phosphoserine aminotransferase (EC 2.6.1.52) (Phosphoserine aminotransferase, putative) | 5.16 |
| 40 | EHI_131880 | -1.66 | M | Acyl-coA synthetase, putative | 3.48 |
| 41 | EHI_105140 | -1.65 | VH | 40S ribosomal protein S15a, putative | 11.23 |
| 42 | EHI_104360 | -1.65 | H | Nucleoside diphosphate kinase, putative | 8.29 |
| 43 | EHI_189510 | -1.65 | M | RhoGAP domain containing protein | 4.53 |
| 44 | EHI_044610 | -1.65 | M | Ribosome biogenesis protein NEP1, putative | 4.53 |
| 45 | EHI_030810 | -1.65 | H | Malate dehydrogenase (EC 1.1.1.37) | 8.73 |
| 46 | EHI_004600 | -1.62 | H | Hybrid-cluster protein (Hydroxylamine reductase) | 7.41 |
| 47 | EHI_110520 | -1.60 | H | Glutamate synthase beta subunit, putative (NADPH-dependent oxidoreductase) | 8.68 |
| 48 | EHI_068120 | -1.60 | M | Ras family GTPase | 4.55 |
| 49 | EHI_073660 | -1.58 | M | Tyrosine kinase, putative | 1.36 |
| 50 | EHI_167270 | -1.58 | VH | 40S ribosomal protein S15a, putative | 11.23 |
| 51 | EHI_050650 | -1.57 | M | Protein kinase, putative | 2.33 |
| 52 | EHI_052770 | -1.56 | M | Glucosidase II alpha subunit, putative | 2.95 |
| 53 | EHI_068090 | -1.55 | M | Serine carboxypeptidase (S28) family protein | 5.41 |
| 54 | EHI_015380 | -1.55 | H | Immuno-dominant variable surface antigen | 7.52 |
| 55 | EHI_169580 | -1.49 | H | Nucleoside transporter, putative | 8.36 |
| 56 | EHI_029560 | -1.49 | H | Glucosamine--fructose-6-phosphate aminotransferase | 7.27 |
| 57 | EHI_146330 | -1.47 | H | Calpain large subunit domain III containing protein | 6.08 |
| 58 | EHI_111790 | -1.47 | M | LIM zinc finger domain containing protein | 4.81 |
| 59 | EHI_198620 | -1.44 | M | PPi-type phosphoenolpyruvate carboxykinase 3 | 3.99 |
| 60 | EHI_127830 | -1.41 | H | Long-chain-fatty-acid--CoA ligase, putative | 6.20 |
| 61 | EHI_195130 | -1.37 | M | Auxin efflux carrier family protein, putative | 5.24 |
| 62 | EHI_103450 | -1.34 | M | Actin binding protein, putative | 4.79 |
| 63 | EHI_000440 | -1.34 | H | UDP-glucose pyrophosphorylase, putative | 6.74 |
| 64 | EHI_069570 | -1.32 | M | Methyltransferase-like protein 2, putative | 4.16 |
| 65 | EHI_051730 | -1.31 | M | Phosphoribulokinase/uridine kinase family protein | 5.98 |
| 66 | EHI_183460 | -1.31 | H | Elongation factor 1 beta, putative | 6.72 |
| 67 | EHI_166490 | -1.31 | H | Alcohol dehydrogenase, putative | 7.72 |
| 68 | EHI_165350 | -1.30 | VH | Malate dehydrogenase, putative | 10.51 |
| 69 | EHI_130930 | -1.27 | VH | Purine nucleoside phosphorylase, putative | 10.47 |
| 70 | EHI_050570 | -1.25 | M | Cysteine proteinase, putative | 5.78 |
| 71 | EHI_105330 | -1.23 | M | Coronin | 4.07 |
| 72 | EHI_010060 | -1.22 | H | RNA recognition motif domain containing protein | 7.65 |
| 73 | EHI_000730 | -1.22 | H | Pyrophosphate--fructose 6-phosphate 1-phosphotransferase | 8.48 |
| 74 | EHI_142150 | -1.21 | M | Battenin | 3.68 |
| 75 | EHI_155730 | -1.20 | M | 1-acyl-glycerol-3-phosphate acyltransferase | 5.36 |
| 76 | EHI_044970 | -1.18 | VH | Malic enzyme (Malic enzyme, putative) | 10.32 |
| 77 | EHI_054690 | -1.17 | H | Metal dependent hydrolase, putative | 6.51 |
| 78 | EHI_156230 | -1.16 | M | Transporter, major facilitator family | 4.49 |
| 79 | EHI_067920 | -1.16 | M | CDP-alcohol phosphatidyltransferase family protein | 4.56 |
| 80 | EHI_010070 | -1.16 | H | Xaa-Pro dipeptidase, putative | 7.65 |
| 81 | EHI_096750 | -1.15 | M | Alanine aminotransferase, putative | 5.41 |
| 82 | EHI_101280 | -1.15 | M | Protein tyrosine kinase domain-containing protein | 3.80 |
| 83 | EHI_062790 | -1.12 | VH | Thioredoxin, putative | 9.20 |
| 84 | EHI_008110 | -1.12 | M | Transporter, major facilitator family | 3.86 |
| 85 | EHI_047730 | -1.10 | M | Glucose-6-phosphate isomerase | 5.63 |
| 86 | EHI_107210 | -1.10 | M | NADP-dependent alcohol dehydrogenase, putative | 5.40 |
| 87 | EHI_094030 | -1.09 | H | Actin binding protein, putative | 7.38 |
| 88 | EHI_100280 | -1.09 | M | Amino acid-polyamine transporter, putative | 4.76 |
| 89 | EHI_103690 | -1.07 | H | Soluble calcium-activated nucleotidase 1, putative | 7.54 |
| 90 | EHI_110810 | -1.06 | M | Unconventional myosin IB | 5.28 |
| 91 | EHI_106090 | -1.06 | M | Starch branching enzyme, putative | 5.52 |
| 92 | EHI_164900 | -1.05 | M | Rab family GTPase | 5.87 |
| 93 | EHI_010690 | -1.04 | M | Rab GTPase activating protein, putative | 3.97 |
| 94 | EHI_152760 | -1.04 | H | Metal cation transporter, zinc (Zn2 )-iron (Fe2 ) permease (ZIP) family (Zinc transporter, putative) | 7.15 |
| 95 | EHI_073600 | -1.03 | H | 40S ribosomal protein S15a, putative | 8.89 |
| 96 | EHI_098210 | -1.03 | H | Lysine and glutamic acid-rich protein 1 (KERP1) | 7.07 |
| 97 | EHI_193360 | -1.03 | H | Histone H3, putative | 7.45 |
| 98 | EHI_110250 | -1.02 | M | Protein kinase domain containing protein | 4.61 |
| 99 | EHI_027700 | -1.02 | H | Molybdopterin cofactor sulfurase, putative | 6.75 |
| 100 | EHI_138750 | -1.00 | M | Tyrosine kinase, putative | 4.34 |
| 101 | EHI_023260 | -0.97 | M | Nicotinate phosphoribosyltransferase, putative | 5.25 |
| 102 | EHI_054530 | -0.97 | M | Serine carboxypeptidase (S28) family protein | 5.95 |
| 103 | EHI_073580 | -0.96 | M | Leucine-rich repeat containing protein | 5.20 |
| 104 | EHI_035800 | -0.94 | M | Ras GTPase activating protein, putative | 4.69 |
| 105 | EHI_191900 | -0.94 | M | Cortexillin, putative | 5.31 |
| 106 | EHI_183400 | -0.93 | H | Galactose-inhibitable lectin 35kD subunit, putative | 6.09 |
| 107 | EHI_111610 | -0.92 | M | Tryptophanase, putative | 5.51 |
