# Supplementary material for: Transcriptomic analysis reveals novel downstream regulatory motifs and highly transcribed virulence factor genes of Entamoeba histolytica
Source: BMC Genomics. 2019 Mar 12;20:206. doi: 10.1186/s12864-019-5570-z (PMC6416950; doi:10.1186/s12864-019-5570-z)
Supplement: Supplementary file 19 — Up regulated genes during serum replenishment (Of the 32 up regulated genes 16 were uncharacterized). (DOCX 15 kb) [file 12864_2019_5570_MOESM19_ESM.docx]

**Additional file 19: Up regulated genes during serum replenishment** (Of the 32 up regulated genes 16 were uncharacterized)

| **S.No** | **Gene** | **Log_2_ (FC)** | **Class** | **Annotation** | **Log_2_ TPM** |
| --- | --- | --- | --- | --- | --- |
| 1 | EHI_095820 | 2.78 | M | ATP-binding cassette, putative | 5.37 |
| 2 | EHI_131880 | 2.45 | M | Acyl-coA synthetase, putative | 3.48 |
| 3 | EHI_197340 | 2.35 | M | Sulfotransferase, putative | 3.86 |
| 4 | EHI_183000 | 1.66 | M | Gal/GalNAc lectin Igl2, putative | 2.16 |
| 5 | EHI_163240 | 1.47 | M | Phosphatidate cytidylyltransferase, putative | 5.93 |
| 6 | EHI_172850 | 1.32 | M | Surface antigen ariel1, putative | 5.00 |
| 7 | EHI_080200 | 1.21 | VH | Surface antigen ariel1, putative | 9.14 |
| 8 | EHI_101280 | 1.15 | M | Protein tyrosine kinase domain-containing protein | 3.80 |
| 9 | EHI_186470 | 1.13 | VH | Surface antigen ariel1, putative | 9.14 |
| 10 | EHI_153680 | 1.09 | M | Carbohydrate degrading enzyme, putative | 4.61 |
| 11 | EHI_099700 | 1.03 | H | NAD(FAD)-dependent dehydrogenase, putative | 6.13 |
| 12 | EHI_062790 | 1.02 | VH | Thioredoxin, putative | 9.20 |
| 13 | EHI_156310 | 1.00 | H | Ribonuclease, putative | 7.82 |
| 14 | EHI_029560 | 0.91 | H | Glucosamine fructose-6-phosphate aminotransferase | 7.27 |
| 15 | EHI_010850 | 0.90 | H | Cysteine proteinase, putative | 8.48 |
| 16 | EHI_115710 | 0.89 | M | Serine/threonine-protein phosphatase | 4.85 |
