# Supplementary material for: Transcriptomic analysis reveals novel downstream regulatory motifs and highly transcribed virulence factor genes of Entamoeba histolytica
Source: BMC Genomics. 2019 Mar 12;20:206. doi: 10.1186/s12864-019-5570-z (PMC6416950; doi:10.1186/s12864-019-5570-z)
Supplement: Supplementary file 20 — Down regulated genes during serum replenishment (Of the 28 down regulated genes 09 were uncharacterized). (DOCX 16 kb) [file 12864_2019_5570_MOESM20_ESM.docx]

**Additional file 20: Down regulated genes during serum replenishment (**Of the 28 down regulated genes 09 were uncharacterized**)**

| **S.No** | **Gene** | **Log_2_ (FC)** | **Class** | **Annotation** | **Log_2_ TPM** |
| --- | --- | --- | --- | --- | --- |
| 1 | EHI_054720 | -3.03 | L | CDP-alcohol phosphatidyltransferase family protein | 0.08 |
| 2 | EHI_092190 | -2.07 | M | 3-ketoacyl-CoA synthase | 2.59 |
| 3 | EHI_120360 | -1.97 | H | Grainin, putative | 8.47 |
| 4 | EHI_148580 | -1.97 | M | Choline/ethanolamine kinase, putative | 3.84 |
| 5 | EHI_130860 | -1.95 | M | Longevity-assurance family protein | 1.49 |
| 6 | EHI_023890 | -1.87 | M | Nuclear movement protein, putative | 5.61 |
| 7 | EHI_154270 | -1.85 | L | Cell division control protein 42, putative | -0.92 |
| 8 | EHI_055680 | -1.59 | M | Heat shock protein, Hsp20 family, putative | 3.03 |
| 9 | EHI_164460 | -1.57 | M | Leucine-rich repeat containing protein | 2.31 |
| 10 | EHI_185240 | -1.35 | M | Long-chain-fatty-acid--CoA ligase, putative | 2.79 |
| 11 | EHI_050290 | -1.33 | M | SGS domain protein | 5.13 |
| 12 | EHI_023300 | -1.22 | M | TPR repeat protein | 3.95 |
| 13 | EHI_056490 | -1.22 | H | 20 kDa antigen, putative | 7.95 |
| 14 | EHI_155570 | -1.16 | M | Glutamyl-tRNA synthetase, putative | 4.44 |
| 15 | EHI_093330 | -1.12 | H | Helix-turn-helix protein, putative | 6.90 |
| 16 | EHI_151260 | -1.04 | M | DnaJ family protein | 4.76 |
| 17 | EHI_193390 | -1.00 | M | Heat shock protein, Hsp20 family, putative | 5.69 |
| 18 | EHI_153710 | -0.95 | L | Methylene-fatty-acyl-phospholipid synthase | 3.24 |
| 19 | EHI_137110 | -0.89 | H | Copine, putative | 6.25 |
