# Supplementary material for: Transcriptomic analysis reveals novel downstream regulatory motifs and highly transcribed virulence factor genes of Entamoeba histolytica
Source: BMC Genomics. 2019 Mar 12;20:206. doi: 10.1186/s12864-019-5570-z (PMC6416950; doi:10.1186/s12864-019-5570-z)
Supplement: Supplementary file 21 — Primer List. (DOCX 16 kb) [file 12864_2019_5570_MOESM21_ESM.docx]

**Additional file 21: Primer List**

| **Experiment** | **Primer Name** | **Primer sequence** |
| --- | --- | --- |
| Real Time | Rcl1RT FP | \| CCCCAAATGAGTAATCGTATGGGAGAAG \| \| --- \| |
|  | Rcl1RT RP | \| GGCAGTTAATATAACACCATATCCAGGA \| \| --- \| |
|  | Nob1RT FP | \| GAAGACCCAAGATTTCCAAAATTAGGGG \| \| --- \| |
|  | Nob1RT RP | \| CATCAACTTTTTCTTTATGCTCTTCTTCTTCTG \| \| --- \| |
|  | Utp24RT FP | \| GAAACACCTAAACAAGTAGTTGTTAGTCCTATTG \| \| --- \| |
|  | Utp24RT RP | \| GTTAGTATCAAGAAGAAGATGATAAGGAGGAC \| \| --- \| |
|  | CPRTFP | CCAATAACAATAAACACTTCACAGCAGTTGAGTC |
|  | CPRTRP | GTCATAGCAGCAAATGGTCCATCTACTGATA |
|  | APRT FP | GCTGCAACAAGAGAAGGAGCTATTCTTTG |
|  | AP RT RP | AGTTCCAAGGAATCCACTAGCTTTACCAC |
|  | Amy RT FP | CAAGGTGGAGTTATTGTGGAGTAGGAG |
|  | Amy RT RP | GAATTATAATTTCCTGCATTTGATGGTGATCCATTTC |
|  | Actin RT FP | GGGAGACGAAGAAGTTCAAGC |
|  | Actin RT RP | ACAGCTCTTGGTGCATCATC |
|  | Ariel RT FP | GGACAACAAGAAAGTGAAGGATTAGGATCAG |
|  | Ariel RT RP | CCTGTTTGACCCTGTGGATTTTGTTCATTTG |
|  | GAPDH FPRT | CTGCCTTGCACCACTTGCTAAAGTTATTAAC |
|  | GAPDH RP RT | TAGCACATGCACATCTACCAGCTCTC |
|  | TMKB1-18 FPRT | TATGACATTCACAAAAGGTGTAGGAACACC |
|  | TMKB1-18 RPRT | GGATATTCTGATTTAGGAAATGGATCTTGCCA |
| Luciferase assay | 14-3-3M5mutFP | AGGTCAGGGGGGGAATAAAGAATAATAAAAGAG |
|  | 14-3-3M5mutRP | CCCCCCTGACCTATGATATTTTTAGAATTATTTCTATTC |
|  | 14-3-3(DM4)RP | CAGGTACCATTCATGTATTGAACCATTTCATCATATCTTT |
|  | 14-3-3 AccRP | CAGGTACCAGCAGCAACTTGTTTCATGTAT |
|  | 14-3-3RP | AGCAACTTGTTTCATGTATTGAACCATTTCATCATATCTT |
|  | 14-3(Del M6)RP | CATTTCATCATATCTTTCAGCAAGTTTAGAAAGGAAGACAC |
|  | Rpl30Xho1FP | CGCTCGAGATTCATTTGTTGTAGTGGTATAAAAG |
|  | Rpl30AccRP | CAGGTACCAGCTTTGGTTCCAAGGACATA |
|  | RPl30(MutM5)RP | TTACCCCTTGACCTTATGTTTTTAATTTTTTTATTTTTGAAGAATG |
|  | RPl30(Mut M5)F | ATAAGGTCAAGGGGTAAAGTAATAAATTGAAATTAATATGAAAC |
|  | Amylase Xho1FP | CGCTCGAGTTTCATTAACTCCGAAAAAAAGAAG |
|  | Amylase AccRP | CAGGTACCTTTATTATTAACTCCATTGGAATTGAC |
|  | Amy In M5RP | CTTCTAACTTTTTTTAGTTCTTATTTAATACAAAAAATATATTGTTTTA |
|  | Amy In M5FP | GAACTAAAAAAAGTTAGAAGAAAATTCAAAAGAAAG |
|  | Amy M4 RP | GCAACAGCACCAAAAGCCAAAGTAACTACAC |
|  | Amy M4 FP | TTGGTGCTGTTGCCAATTTGATGATGCCAC |
